# Making Potent CAR T Cells Using Genetic Engineering and Synergistic Agents

**DOI:** 10.3390/cancers13133236

**Published:** 2021-06-29

**Authors:** Chi Hoon Park

**Affiliations:** 1 Therapeutics & Biotechnology Division, Korea Research Institute of Chemical Technology, 141 Gajeong-ro, Daejeon 34114, Korea; chpark@krict.re.kr; Tel.: +82-42-860-7416; Fax: +82-42-861-4246; 2 Medicinal & Pharmaceutical Chemistry, Korea University of Science and Technology, Daejeon 34113, Korea

**Keywords:** CAR T cell, genetic engineering, synergistic agents, cancer

## Abstract

**Simple Summary:**

Although CAR T cells are regarded as a revolutionary drug in cancer treatment, however, it still has many limitations. To overcome the disadvantages of CAR T cell, many researchers have tried to optimize the CAR gene. In this review, I summarized the current studies regarding genetic engineering of CAR and synergistic agents to enhance the CAR T cell efficacy both in preclinical and clinical models.

**Abstract:**

Immunotherapies are emerging as powerful weapons for the treatment of malignancies. Chimeric antigen receptor (CAR)-engineered T cells have shown dramatic clinical results in patients with hematological malignancies. However, it is still challenging for CAR T cell therapy to be successful in several types of blood cancer and most solid tumors. Many attempts have been made to enhance the efficacy of CAR T cell therapy by modifying the CAR construct using combination agents, such as compounds, antibodies, or radiation. At present, technology to improve CAR T cell therapy is rapidly developing. In this review, we particularly emphasize the most recent studies utilizing genetic engineering and synergistic agents to improve CAR T cell therapy.

## 1. Introduction

Researchers have tried to harness the immune system to attack tumors for several decades. The first attempt involved the use of heat-killed bacteria by William Coley, a surgeon [1]. After reading that tumor disappeared after a bacterial infection, he injected a mixture of heat-killed bacteria of species *Streptococcus pyrogenes* and *Serratia marcescens*, called Coley’s toxins, to sarcoma patients. Although his work remained controversial, he is regarded as the “Father of Cancer Immunotherapy” for his scientific contribution. In the 1980s, Rosenberg SA in NCI reported that tumor-infiltrating lymphocytes (TILs) were effective in eradicating tumors [2]. TILs were extracted from tumor lesions, expanded ex vivo, and then re-infused together with interleukin-2 (IL-2) into the patient. Although the manufacturing process is complicated, the clinical trial of TILs therapy is still active on melanoma [3].

In late 1980s, Eshhar Z in Weizmann institute suggested a fancy T cell model using an artificial receptor composed of a constant domain of the T-cell receptor fused to the variable domain of the antibody [4]. With the help of the variable domain of an antibody, these genetically modified T cells were redirected to antigen-expressing tumors. A chimeric receptor composed of a single chain fragment variable (scFv) and a signal transducing domain, which resembles the current first generation chimeric antigen receptor (CAR), was reported in 1993 [5]. In preclinical models, CAR T cells showed antigen-specific tumor elimination in vitro and in vivo [6,7,8,9]. In the early 2010s, complete remission with anti-CD19 CAR T cell therapy was reported in patients with chronic lymphoblastic leukemia (CLL) and acute lymphoblastic leukemia (ALL) [10,11]. The results were so overwhelming that researchers praised CAR T cell therapy with enthusiasm. Currently, two CAR T cell products, Kymriah and Yescarta, are approved by the US FDA for the treatment of pediatric ALL and adult large B-cell lymphoma (LBCL) [12]. However, it is still challenging to fabricate CAR T cells that work successfully on some types of leukemia, such as T cell lymphoma and acute myeloid leukemia (AML), and most solid tumors [13,14,15].

The CAR T cells currently under development have been engineered genetically to compensate for the weaknesses of existing CAR T cells. To overcome the obstacles of CAR T cell therapy, such as poor activity in solid tumors and severe side effects caused by activated CAR T cells, the current CAR constructs have been modified in multiple ways to express specific genes or to deplete immune-suppressive genes. In addition, compounds or radiation have been applied to CAR T cell therapy to synergize its effect. The ex vivo culture of CAR T cells has also been studied intensively to maximize the therapeutic effect of CAR T cells. This review is intended to provide a comprehensive overview for individuals who wish to invent potent CAR T cells using genetic engineering or combination agents.

## 2. CAR Construct

### 2.1. Basic Design of CAR

**Figure 1:**
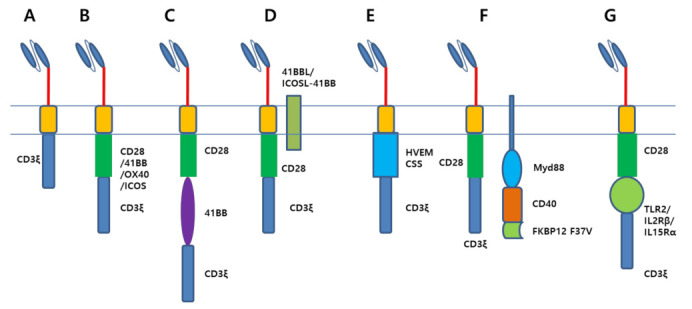
Various constructs of chimeric antigen receptor. (**A**). First-generation CAR construct is composed of a single chain variable fragment fused with a CD3ζ endodomain. (**B**). Second-generation CAR construct has a costimulatory domain including CD28, 4-1BB, OX40, or ICOS. (**C**). Third-generation CAR construct has two costimulatory domains. (**D**). Coexpression of ligand of immune activating receptors, such as 41BBL or ICOSL-41BB, enhanced the anti-tumor effect of CAR T cell. (**E**). Recent report demonstrated that herpes virus entry mediator (HVEM) co-stimulatory signal domain (CSS) can be used for CAR constructs. (**F**). Myd88/CD40 signaling greatly potentiated the CAR activity in preclinical models. Myd88/CD40 signaling can be modulated by dimerization of FKBP12 F37V domain. (**G**). Toll-like receptor (TLR) domain and cytokine receptor domains (IL2Rβ or IL15Rα) were used as co-stimulatory domain to increase CAR T cell effect.

After molecular cloning of the CD3ζ clone [16], Eshhar et al. developed a CAR construct composed of a scFv domain fused to the CD3ζ domain [5]. This first-generation CAR T cell showed antigen-specific T cell activation in vitro (Figure 1A); however, the in vivo efficacy was modest [17]. The second-generation CAR construct was incorporated with CD28 domain and showed an enhanced ability to produce cytokine in CAR T cells (Figure 1B) [18]. Moreover, the proliferation of CAR T cell was improved by CD28-mediated signaling [19], and the cooperation of CD3ζ signaling and CD28 signaling improved CAR T cell activation [20,21]. In NOD.Cg-Prkdcscid Il2rgtm1Wjl/SzJ (NSG) mice models, CAR T cells with CD28 signaling had a much better antitumor effect than CAR T cells without CD28 signaling [22,23,24,25]. In clinical experiments, CD28 co-stimulation was proven to be important for T cell expansion and in vivo persistence in lymphoma patients [26]. In 2004, another costimulatory domain, 4-1BB, was introduced to CAR T cells, which enhanced the in vivo persistence of CAR T cells, leading to a superior antitumor effect in preclinical models [24,27]. In 2009, the June group first reported the third-generation CAR construct encoding both the CD28 and 4-1BB cytoplasmic domains as costimulatory domains (Figure 1C) [25]. After that, various third-generation CAR T cells, which utilized OX40 or inducible T-cell costimulatory**** (ICOS) as the second costimulatory domain, were developed [25,28,29,30,31,32,33,34]. Some reports claimed that third-generation CAR T cells persist longer than second-generation CAR T cells in vivo [35]. However, it is controversial whether the antitumor efficacy of third-generation CAR T cells is superior to second-generation CAR T cells. In a preclinical model, second-generation CAR T cells, including 4-1BB-based (bbz) or CD28-based (28z) CAR T cells, and third-generation CAR T cells, such as 4-1BB- and CD28-based CAR T cells (28bbz), showed a similar antitumor effect, whereas CAR T cells with only CD3ζ (z) showed much weaker effects than others [25]. However, other reports claimed that the combination of 4-1BB signaling and CD28 signaling enhanced the in vivo persistence of CAR T cells, leading to a superior anti-leukemic effect [33,34]. Researchers have also tried to reveal the best costimulatory domain for CAR T cell efficacy. 4-1BB co-stimulation of CAR T cells promotes longer persistence, greater ex vivo expansion, and better survival than CD28 co-stimulation [36]. In addition, 4-1BB signaling ameliorates T cell exhaustion by CAR tonic signaling, which is a signaling in an antigen-independent manner due to the CAR clustering, leading to a superior antitumor effect [37]. In an NSG mouse xenograft model, the ICOS-incorporated 4-1BB-based CAR construct—ICOS-BBz—showed a superior antitumor effect over BBz-based CAR construct by enhancing CAR T cell persistence [38]. Another study reported that CD28-based CAR T cells with 4-1BB exhibited higher in vivo efficacy than CAR T cells with OX40 [39]. Overall, it is regarded that CD28-based CAR T cells are CD45RO^+^ CCR7^−^, the same as effector memory T cells, and 4-1BB-based CAR T cells are CD45RO^+^ CCR7^+^, the same as central memory T cells. Therefore, 4-1BB CAR T cells are pushed toward a long-lasting central memory response, and CD28 CAR T cells are pushed toward a short-lived effector response [40,41]. However, in clinical studies comparing 28z and 4-1bbz, their antitumor effect was almost same, although bbz CAR T cells exhibited a more favorable safety profile than 28z [42,43]. Furthermore, it has been revealed that first-generation CAR (19z) T cells also eliminate the patient tumor as efficiently as second-generation CAR (1928z) T cells, even though second-generation CAR T cells persist for longer periods than first-generation CAR T cells in patients [44].

### 2.2. A Novel Design of CAR

Several groups have developed CAR T cells coexpressing ligand proteins of immune activating receptors, such as 41BB ligand (41BBL) or ICOS ligand (ICOSL), to produce costimulatory signalings (Figure 1D) [45,46]. Anti-CD38 CAR T cells expressing 41BBL exhibited a potent antitumor effect with improved proliferative capacity and enrichment of central memory T cells in an NSG xenograft model. [45] ICOSL or ICOSL-41BB expression also reinforced the expansion capability of CAR T cells and reduced the apoptotic events of CAR T cells [46]. A new costimulatory domain, the herpes virus entry mediator (HVEM) cytoplasmic domain, was incorporated into the CAR construct (Figure 1E) [47]. HVEM-based CAR T cell showed enhanced effector function, balanced differentiation into effector memory and central memory subsets, and reduced T cell exhaustion leading to superior antitumor effect in comparison to CD28-based or 41BB-based CAR T cells. The Spencer group used a completely different costimulatory domain, Myd88/CD40 (Figure 1F). Myd88 is the adaptor molecule for the IL-1 receptor family and Toll-like receptor (TLR) signaling. CD40 is a T cell costimulatory molecule found on antigen-presenting cells. They designed a remote control system to turn on the Myd88/CD40 signaling through a compound. Myd88/CD40 signaling enhanced the cytolytic activity and proinflammatory cytokine secretion of CAR T cells and reduced the surface expression of PD-1 on CAR T cells. As a result, CAR T cells with Myd88/CD40 signaling demonstrated a superior antitumor effect over CD28-based CAR T cells in an NSG muse model [48]. This system is also applicable to CAR-Natural Killer (NK) cells [49]. The Toll/interleukin-1 receptor (TIR) domain of the Toll-like receptor, which plays a significant role in the innate immune system, was also incorporated into the CAR construct, and it potentiated the CAR T cells antitumor efficacy both in preclinical and clinical trials (Figure 1G) [50,51,52]. Kagoya et al. invented a second-generation CAR construct encoding additional truncated IL2 receptor β-chain (IL2Rβ) and a signal transducer and activator of transcription proteins (STAT) 3/5 binding motif (YXXQ) at the C terminus of CD3ζ [53]. These CAR T cells produced all three signals, including T cell receptor (TCR), CD28, and cytokines, which are required for T cell activation. In preclinical models, these CAR T cells displayed increased proliferation and attenuated terminal differentiation, which leads to an enhanced antitumor effect. Nair et al. developed a CAR construct encoding signaling motif of IL15Rα [54]. IL15Rα co-signaling exhibited enhanced T cell expansion and cytotoxic activity.

### 2.3. Transmembrane and Hinge Domains of CAR

Recently, it was found that hinge and transmembrane (TM) domains are essential for CAR T cell activation. T cells expressing CARs with CD8α hinge and transmembrane domain produced fewer cytokines and exhibited lower levels of activation-induced cell death compared to those with CD28 hinge and transmembrane domain, resulting in enhanced in vivo efficacy [55]. A successful clinical trial was performed using CAR T cells with a hinge domain to produce low levels of cytokines, and high levels of anti-apoptotic molecules [56]. The hinge and transmembrane domains of CAR determine the CAR expression level, which correlates with antitumor efficacy [57]. Several groups have used the mutant CH2CH3 domain of IgG4 (L235E, N297Q) for the CAR hinge domain to escape FcγR recognition by the innate immune system [58,59]. Interestingly, ICOS transmembrane has a unique ability to promote association with lymphocyte-specific protein tyrosine kinase (Lck) and subsequent phosphatidylinositol 3-kinases (PI3K) activation. Based on this, ICOS TM was incorporated into the CAR construct, which facilitated the interaction between CAR T cells and target cells [60].

### 2.4. CAR Contructs Designed to Exhibit Reduced T Cell Activiation Lead to the Superior Antitumor Activity

**Figure 2:**
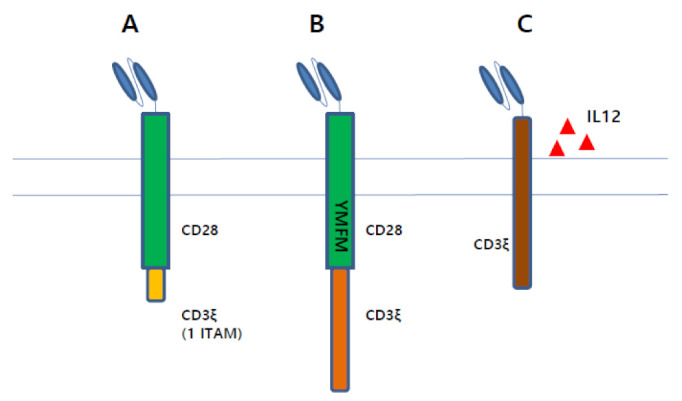
CAR constructs designed to exhibit reduced T cell activation are likely to achieve enhanced preclinical results. (**A**). A CAR construct containing single ITAM in CD3ζ endodomain demonstrates excellent anti-tumor effect. (**B**). Defect in binding with Grb2 by mutant encoding YMFM instead of YMNM in CD28 endodomain reduced T cell exhaustion, which leads to excellent anti-tumor effect. (**C**). First-generation CAR T cells secreting IL12 is superior to second-generation CAR T cells.

Recent CAR T cell studies demonstrated that CAR T cells designed to exhibit reduced activation are likely to achieve excellent preclinical results. CD3ζ has three immunoreceptor tyrosine-based activation motifs (ITAMs), which contributes to the downstream signalings in T cells. Although ITAM-mediated signalings are critical for T cell activation, they can also trigger T cell differentiation and exhaustion [61]. Feucht et al. developed CAR T cells with only one ITAM in the CD3ζ domain, and these CAR T cells exhibited far superior preclinical results over those with three ITAMs in the CD3ζ domain by balancing memory and effector programs (Figure 2A) [62]. Guedan et al. found that changing asparagine of CD28 (YMNM) to phenylalanine (YMFM) promoted the antitumor activity of CAR T cells (Figure 2B) [63]. As asparagine is important for CD28 interaction with growth factor receptor-bound protein 2 (Grb2), which is responsible for the nuclear factor of activated T cells (NFAT) activation and IL-2 production, CD28 with YMFM resulted in reduced cytokine production, subsequently leading to reduced T cell differentiation and exhaustion. In a syngeneic mouse model, anti-CD19 CAR T cells expressing IL12 with only CD3ζ signaling were shown to be superior over IL12 expressing CAR T cells with both CD3ζ and costimulatory signaling (Figure 2C) [64]. Thus, it can be said that optimized CAR T cell activation is critical for excellent CAR T cell activity.

### 2.5. Antigen-Binding Domains of CAR

**Figure 3:**
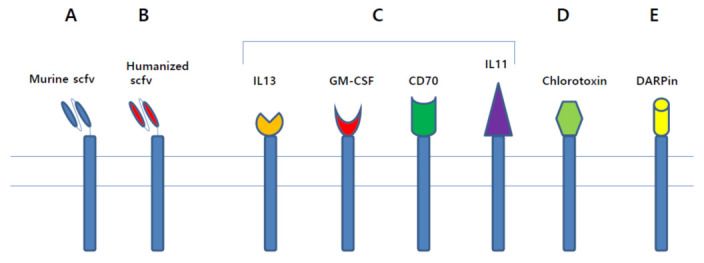
Various antigen binding domains. (**A**) In early days of CAR T cell research, murine scFv was used as antigen binding domain. (**B**) To avoid disadvantages of murine scFv, humanized scFv was used in recent research. (**C**) Natural ligands, such as IL13, GM-CSF, CD70, and IL11, can be used for antigen binding domain. (**D**) As chlorotoxin can bind to most primary glioblastoma cells, toxin-based CAR T cells were developed. (**E**) Designed ankyrin repeat proteins (DARPin), which are composed of repeating ankyrin motifs, can be used for antigen binding domain.

The standard approach for the antigen binding domain in developing CAR T cell is to use single-chain variable fragment of monoclonal antibody. Murine scFvs were widely used in the early days of CAR T cell research, and both CAR T cell products approved by the US FDA—KYMRIAH and YESCARTA—use the same murine scFv, FMC63 (Figure 3A). However, cellular immunity specific to peptides of murine scFv, resulting in reduced expansion and suppressed antitumor effect of CAR T cells, has been reported in patients [65,66]. Moreover, murine scFv-based CAR T cells sometimes caused the development of anaphylaxis [67]. To avoid these disadvantages of murine scFv, fully human scFv, which can be generated by transgenic mice or phage display, or humanized scFv-based CAR T cells have been extensively studied (Figure 3B) [55,68,69,70,71,72,73,74]. CAR T cells with humanized scFv showed successful results in clinical trials [75,76,77,78]. Another approach for the antigen-binding domain is to use naturally existing ligands (Figure 3C). IL13Rα is known to be overexpressed in the majority of glioblastoma tumors, not in normal brain tissue [79,80]. IL13-based CAR T cells, which are capable of recognizing IL13Rα, showed excellent efficacy in preclinical models [81,82]. Intracranial infusions of IL13-based CAR T cells induced the regression of tumors in glioblastoma patients [83,84]. Another example includes granulocyte-macrophage colony-stimulating factor (GM-CSF)-based CAR T cells targeting CD116, GM-CSF receptor, for the curative treatment of juvenile myelomonocytic leukemia (JMML). These CAR T cells have been shown to inhibit the proliferation of JMML CD34^+^ cells, but not normal CD34^+^ cells [85]. CD70 CAR T cells are other examples of ligand-based CAR T cells [86,87,88]. IL11-based CAR T cells were found to be effective against IL11Rα overexpressing osteosarcoma cells in a mouse model [89].

Wang et al. paved the way to the use of toxins for the antigen-binding domain. As chlorotoxin, which is a 36-amino acid peptide found in the venom of the deathstalker scorpion, binds specifically to glioblastoma cells, not normal cells, chlorotoxin-based CAR T cells were invented and studied for their anti-cancer effect (Figure 3D) [90]. It has been demonstrated that chlorotoxin binds to most primary glioblastoma tumors, whereas antibodies to Her2, IL13Rα2, or EGFR binds to a small population of primary glioblastoma tumors. Furthermore, an in vivo xenograft assay demonstrated that chlorotoxin-based CAR T cells mediated complete tumor eradication.

Designed ankyrin repeat proteins (DARPin) can also be used for the antigen binding domain (Figure 3E). DARPins, which are composed of repeating ankyrin motifs, are known to be small in size, thermodynamically stable, less immunogenic and less prone to aggregate compared to scFvs [91]. CAR T cells designed with anti-Her2 DARPin or DARPins specific for Her2, epidermal growth factor receptor (EGFR), and epithelial cell adhesion molecule (EpCAM) showed potent tumor regression in preclinical models [92,93,94].

## 3. Genetic Engineering to Express or Delete Specific Proteins

### 3.1. Expression of Specific Proteins

An enhanced antitumor effect can be achieved by expressing or depleting specific proteins in CAR T cells (Table 1). CAR T cells expressing telomerase reverse transcriptase (TERT) have been designed [95]. As shortening of telomeric DNA is correlated with cell senescence, CAR T cells expressing TERT exhibited prolonged in vivo persistence, and superior antitumor efficacy in NSG mice models. Another approach is to target the tumor microenvironment (TME). Heparanase, which is secreted by immune cells, is an important protein for degrading heparan sulfate proteoglycans, one of the main extracellular matrix components [96,97]. Heparanase expressing CAR T cells showed improved tumor infiltration, leading to enhanced antitumor activity [98]. Lynn et al. invented c-Jun expressing CAR T cells. They found that T cell exhaustion was associated with defects in IL-2 production, and that IL-2 production was driven by activator protein (AP-1) heterodimer, c-Fos-c-Jun. The c-Jun expressing anti-GD2 CAR T cells exhibited greatly enhanced antitumor activity [99].

### 3.2. Depletion of Specific Proteins

Contrary to the above-mentioned approach, some researchers have achieved enhanced antitumor activity by depleting specific genes (Table 1). Chen J. et al. analyzed RNA sequencing data from CAR T cells and found that the nuclear receptor subfamily 4A (NR4A) is involved in the expression of the inhibitory receptors, programmed cell death protein 1 (PD1) and T cell immunoglobulin and mucin domain-containing protein 3 (TIM3). CAR T cells depleted of three NR4A genes promoted tumor regression [131]. The fact that Tet methylcytosine dioxygenase 2 (TET2) depletion improves the antitumor efficacy of CAR T cells was discovered accidentally. In a clinical study, Fraietta et al. found that anti-CD19 CAR T cells in one leukemic patient, who experienced complete remission, originated from one single T cell in which the TET2 gene was disrupted by lentiviral vector-mediated CAR gene integration into 4q24 loci [132]. They proved that TET2 depletion drives CAR T cells into a central memory phenotype. Protein phosphatases were also targeted for depletion in CAR T cells. Protein tyrosine phosphatase N2 (PTPN2) is known to suppress TCR signaling by dephosphorylating Lck [133]. Depletion of phosphotyrosine phosphatase, non-receptor type 22 (PTPN22) in CD8^+^ T cells is known to enhance their capacity to eradicate tumors [134]. Enhanced tumor rejection was achieved by CAR T cells depleted with PTPN2 or PTPN22 [135,136]. As elevated levels of cholesterol triggers the proliferation and cytotoxic activity of CD8^+^ T cells, the cholesterol esterification enzyme was also studied to enhance CAR T cell activity [153,154]. The activity of the cholesterol esterification enzyme was suppressed by either knocking down or using the inhibitor [140,141]. As expected, CAR T cells lacking cholesterol acyltransferase 1 showed improved antitumor effects in preclinical models. Some researchers have focused on A2a receptor for combination therapy with CAR T cells. A2aR binds to adenosine and leads to increased production of intracellular cyclic adenosine monophosphate (cAMP), which suppresses T cell activation [155,156]. A2aR in CAR T cells was upregulated by stimulation with target cancer cells. A2aR depletion by shRNA, when combined with PD1 blockade, increased CAR T cell efficacy in a syngeneic mouse model [152].

### 3.3. Inhibition of TGF-β Signaling

As transforming growth factor-beta (TGF-β) is one of the important immunosuppressive cytokines, removing TGF-β signaling from CAR T cells could lead to enhanced antitumor efficacy (Table 1). TGF-β signaling in CAR T cell can be suppressed by using oncolytic adenovirus targeting TGF-β, genome-edited technology, expressing dominant-negative TGF-β receptor, or TGF-β inhibitors [137,138,139,157]. CAR T cells lacking TGF-β signaling showed an enhanced antitumor effect. SD-208, a kinase inhibitor that blocks TGF-β signaling, enhances the antitumor effect of CAR T cells by increasing the viability of CAR T cells and suppressing PD-1 expression [137]. Knocking out TGF-β receptor II (TGFBR2) reduced TGF-β signaling and suppressed T_reg_ conversion and exhaustion of CAR T cells [138]. Blocking TGF-β signaling by expressing dominant negative TGF-β receptor II enhanced cytokine secretion and increased CAR T cell proliferation [139].

### 3.4. Genome Editing Technology in CAR T Cell

Clustered regularly interspaced short palindromic repeats (CRISPR)-Cas9 technology is actively applied to CAR T cells to enhance antitumor activity. Contrary to retroviral integration, CRISPR-Cas9 technology enables the CAR gene to be targeted into a specific locus in the genome. Eyquem et al. targeted the anti-CD19 CAR gene to the T-cell receptor alpha chain (TRAC) locus through CRISPR/Cas9. The surface CAR expression of genome-edited CAR T cells is known to be regulated by antigen stimulation. In addition, these CAR T cells have relatively lower expression of immune inhibitory receptors, such as PD1, lymphocyte-activation gene 3 (LAG3), and TIM3. As a result, these TRAC-targeted CAR T cells exhibited superior antitumor efficacy over retrovirally encoded CAR T cells in a preclinical model [158].

Diacylglycerol kinase(DGK) inhibition is known to reinvigorate the function of hypofunctional tumor-infiltrating immune cells [159]. Based on this knowledge, Toolgen Inc. used CRISPR/Cas9 to develop anti-EGFRvIII CAR T cells depleted with DGK. These CAR T cells showed increased TCR signaling, leading to excellent efficacy in preclinical studies [142].

### 3.5. Inhibition of PD-1 Signaling

**Figure 4:**
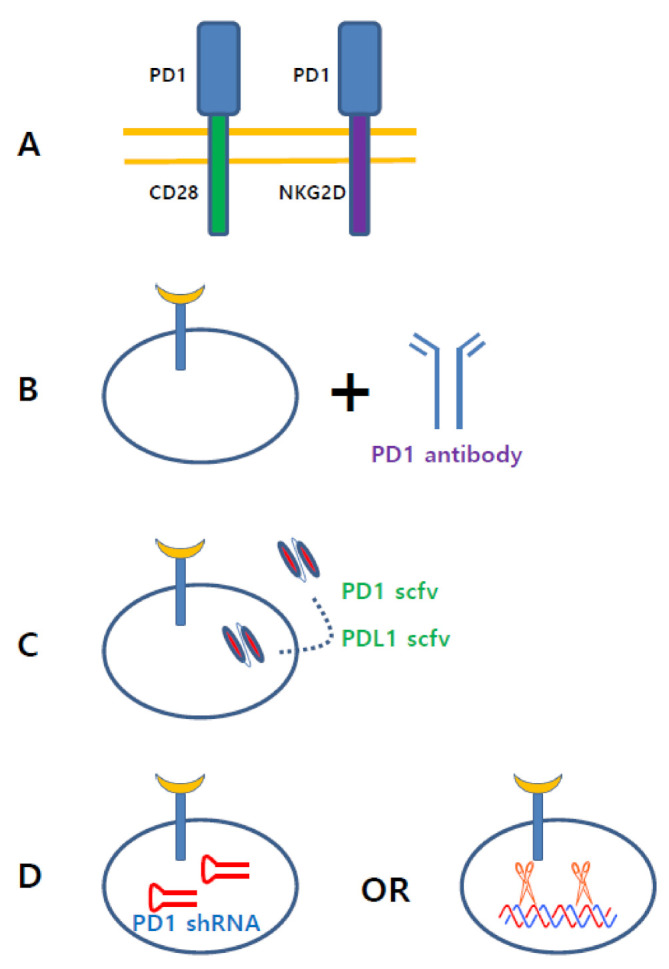
Various approaches targeting PD1 signaling in CAR T cell. (**A**). Decoy receptors, composed of PD1 extracellular domain fused with CD28 or NKG2D endodomain, convert immune inhibitory signal to immune activating signal. (**B**). CAR T cell therapy combined with PD1 antibody demonstrated excellent anti-cancer effect in preclinical models. (**C**). CAR T cells secreting PD1 scFv or PDL1 scFv potentiates CAR T cell activity. (**D**). Depletion of PD1 protein by shRNA or CRISPR technology increased anti-cancer activity of CAR T cell.

In genome-editing research of CAR T cells, immune suppressive receptors have been the most focused (Figure 4). PD-1 or Lag3 knockout CAR T cells by CRISPR-Cas9 exhibited excellent in vivo efficacy [143,160]. The PD1 expression level in CAR T cells has been regarded as a significant factor in determining the efficacy of CAR T-cell therapy. Clinical and preclinical studies have demonstrated that PD1 expression in CAR T cells is negatively associated with clinical outcome [161,162,163]. To downregulate the inhibitory signaling from PD1, some groups developed a smart system to use decoy PD1 receptors (Figure 4A). They made artificial proteins composed of the extracellular domain of PD1 fused to the cytoplasmic domain of CD28, which converted the immune suppressive signal to immune activating signal. Preclinical data showed that this decoy PD1 receptor enhanced the antitumor effect of CAR T cells by decreasing tumor-induced hypofunction and enhancing the tumor infiltration of CAR T cells [144,145,146,147]. The Barber and Zhu group developed chimera protein composed of PD1 extracellular domain fused to DNAX-activating protein 10 (Dap10) or natural killer group 2 member D (NKG2D) [148,149,150,151]. These PD1-based CAR T cells directly recognized and eradicated cancer cells expressing PD1 ligands, such as PDL1 or PDL2. PD1-based inhibitory chimeric antigen receptors have also been developed, in which the antigen-binding domain was fused with the PD1 or cytotoxic T lymphocyte antigen 4 (CTLA4) cytoplasmic domain to divert off-target response [164].

It is well known that PD1 antibodies exhibit excellent clinical efficacy against numerous tumors [165]. Combination therapies of PD1 antibodies and CAR T cells have been actively studied in preclinical and clinical studies to achieve a synergistic effect (Figure 4B) [78,166,167,168,169,170,171,172,173]. Since PD1 expression in CAR T cells is elevated by PDL1 positive tumor cells, PD1 antibody treatment enhanced the antitumor activity of CAR T cells [167]. A case study showed a long term-response to combined PD1 antibodies and anti-CD19 CAR T-cell therapy in a patient with DLBCL [78]. In a humanized mouse model, PD-1 blockade by PD1 antibody showed a synergistic effect with CD28-based CAR T cells lacking the lck binding moiety [166]. PD-1 blockade increased the number of tumor infiltrating anti-EGFR variant III (EGFRvIII) CAR T cells leading to enhanced antitumor activity [171]. This synergistic effect can be achieved using different approaches. Synthetic biology technology has enabled the development of CAR T cells secreting PD1 antibody or PDL1 antibody (Figure 4C) [100,101,102]. In preclinical models, CAR T cells engineered to secrete PD1 scFv demonstrated superior antitumor efficacy compared to combined therapy with a checkpoint inhibitor and CAR T cells. Anti-Glypican 3 (GPC3) CAR T cells expressing a soluble PD1 protein showed a superior antitumor effect against hepatocellular carcinoma [103]. In addition, PD1 depletion by CRISPR/Cas9 or shRNA is an alternative method to achieve PD1 blockade (Figure 4D) [174,175,176,177,178,179,180]. In PD1-depleted CAR T cells, genes involved in DNA replication and cell proliferation were downregulated, whereas genes associated with cell metabolism were upregulated [174]. PD1 depleted CAR T cells produced elevated cytokines and exhibited superior cytotoxicity against PDL1-expressing cancer cells, leading to augmented antitumor activity [176]. A superior antitumor effect of CAR T cells has been obtained by knocking down triple genes, including PD-1, Lag-3, and TIM-3 [181]. Triple inhibitory receptor knockdown upregulated CD56, which contributes to cell–cell adhesion through homophilic interaction and enhanced tumor infiltration, IFNγ secretion, and survival of CAR T cells. PD1 expression can be modulated by targeting glycosylation, because glycosylation of PD1 is indispensable for PD1 stability in cells. It has been demonstrated that CAR T cell cytotoxicity is enhanced by inhibiting PD1 glycosylation [182].

### 3.6. Chemokine Receptors

Chemokine receptors have also been studied intensively for developing enhanced CAR T cell therapy. It is well known that tumor cells or stromal cells in the tumor microenvironment secrete chemokines such as C-C motif chemokine ligand 2 (CCL2), C-X-C motif chemokine ligand 1 (CXCL1), CXCL2, and CXCL5 [183,184,185]. As chemokines play a key role in T cell trafficking, chemokine receptors have been speculated to contribute to T cell recruitment into the tumor. Several preclinical studies have demonstrated that CAR T cells expressing chemokine receptors, including C-X-C motif chemokine receptor 1 (CXCR1), CXCR2, CCR2, CCR4, and C-C chemokine receptor type 2 (CCR2b), showed enhanced trafficking to tumor lesions and superior antitumor efficacy (Table 1) [104,105,106,107,108,109]. The chemokine receptor, such as CXCR1, also enhanced the trafficking of CAR-NK cells to tumor lesion, subsequently augmenting the anti-tumor response [104]. Jin et al. published an interesting report that CAR T cell trafficking to tumor was enhanced by local X-ray irradiation. As X-rays induced IL-8 cytokine secretion from tumor cells, CAR T cells expressing CXCR2, which is a receptor for IL-8, migrated to tumor lesions irradiated by X-ray, leading to enhanced antitumor effects [110].

### 3.7. Interleukins

Cytokines are the most actively studied proteins in CAR T-cell research. Researchers found that cytokine-secreting CAR T cells exhibited a much superior antitumor effect to normal CAR T cells. These CAR T cells are referred to as TRUCKs, which means T cells redirected for antigen-unrestricted cytokine-initiated killing. The cytokines utilized in TRUCKs are IL12 [111,112,113,114,115,116,117,182], IL18 [118,119,120], IL15 [49,121,122,123], IL21 [124], IL23 [125], IL36γ [126], and IL7 [127] (Table 1). IL12 is known to play a key role in bridging innate and adaptive immunity. In both syngeneic and NSG xenograft mice models, IL12 secreting CAR T cells exhibited enhanced tumor infiltration, expansion, and longer persistence to prolong survival. IL18, which is produced by macrophage, stimulates interferon gamma (IFNγ) secretion in T cells. IL18-secreting CAR T cells not only exhibited long persistence and enhanced in vivo expansion, but also modulate the tumor microenvironment to increase M1 macrophages and NK cells and decrease suppressive CD103^+^ dendritic cells and M2 macrophages. IL15 is known to be crucial for generating T-memory stem cells, and its signaling is mediated by membrane-bound complex IL15/IL15Rα (mbIL15). MbIL15 coexpression increased the population of CD45RO^neg^CCR7^+^CD95^+^, which is similar to T_scm_, to enhance the antitumor effect through the long-term persistence of CAR T cells [122]. In tumor growth, the role of IL-23 is controversial. Recent studies have reported that IL23 is upregulated in tumors to enhance angiogenesis and reduces CD8 T-cell infiltration [128,129]. Prostate-specific membrane antigen (PSMA)-targeted CAR T cells expressing an IL23 antibody eradicated prostate tumor in a preclinical model. [130]. On the contrary, IL-23 engineered CAR T cells showed a promising result in solid tumor models [125]. CAR T cells secreting IL-36γ showed enhanced expansion and prolonged in vivo persistence to produce superior tumor eradication compared to conventional CAR T cells [126]. IL-7 improved the CAR T cells infiltration and survival in the tumor lesion [127].

### 3.8. Artificial Cytokine Receptors

**Figure 5:**
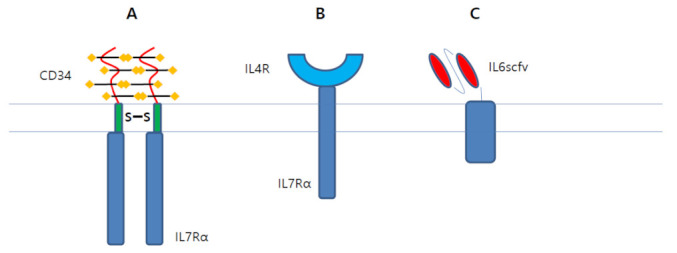
Artificial cytokine receptors potentiate CAR T cell activity. (**A**). C7R, which is composed of CD34 extracellular domain fused to IL7Ra transmembrane and endodomain with inserted disulfide bridge, produces active STAT5 signaling in CAR T cell. (**B**). 4/7 ICR, which is composed of IL7 receptor endodomain fused to IL4 receptor exodomain, convert immune-suppressive IL4 signaling to immune-stimulatory IL7 signaling. (**C**). mbaIL6, which is composed of IL6 scFv fused with transmembrane anchoring peptide, depleted IL6. Thus, it is anticipated that CAR T cells expressing mbaIL6 might prevent CRS.

Recombinant cytokines produced by CAR T cells might cause significant toxicity in the body. To avoid side effects caused by excessive cytokines, several groups developed CAR T cells expressing artificial cytokine receptors [186,187]. As IL7 signaling primarily promotes T cell survival, Shum et al. developed a constitutively active IL7 receptor (C7R) composed of a CD34 extracellular domain fused to IL7Rα transmembrane and endodomain with an inserted disulfide bridge (Figure 5A). C7R expressing CAR T cells induced STAT5 signaling to significantly enhance antitumor effect in vivo [186]. By using the chimeric cytokine receptor, CAR T cells can invert immunosuppressive signaling to activate T cells [188,189]. Mohammed et al. invented 4/7 ICR composed of IL-7 receptor endodomain fused to IL-4 receptor exodomain (Figure 5B). 4/7 ICR convert immune-suppressive IL4 signaling to immune-stimulatory IL7 signaling. CAR T cells expressing 4/7 ICR showed superior antitumor effects in vivo under immune suppressive conditions. Tan et al. invented a CRS protection system by co-expressing non-signaling membrane-bound IL6 receptor (mbaIL6) in CAR T cells [190]. IL6 is known to be central to CRS pathogenesis. MbaIL6 is composed of scFv of the IL6 antibody fused to a transmembrane anchoring peptide (Figure 5C). In vitro and in vivo experiments have demonstrated that CAR T cells expressing mbaIL6 remove IL6. Thus, it is anticipated that CAR T cells expressing mbaIL6 prevent cytokine release syndrome (CRS) without a significant loss of anticancer activity.

## 4. Synergistic Effects of CAR T Cell Therapy with Compounds

### 4.1. Compounds Enhancing CAR T Cell Activity

Many attempts have been made to enhance CAR T cell activity by using small molecules. Several studies have shown the synergy effects of small molecules, including tyrosine kinase inhibitors, cytotoxic agents, histone deacetylase inhibitors, proteasome inhibitors, and immune-modulators, with CAR T cells (Table 2) [191,192,193,194,195,196,197,198]. Afatinib, a tyrosine kinase inhibitor, increased CAR-NK cell infiltration into the tumor [192]. The addition of lenalidomide, an immunomodulatory drug, to CAR T cells cultured ex vivo increased cytotoxicity, cytokine production, and memory phenotype of CAR T cells [193]. Bortezomib, a proteasome inhibitor, augmented the CAR T cell’s antitumor effect both in vitro and in vivo [195]. In a model of Ewing sarcoma, which is characterized by the aberrant expression of hepatocyte growth factor (HGF), AMG102, an HGF receptor neutralizing antibody, improved the antitumor activity of anti-GD2 CAR T cells by increasing CAR T cell trafficking into the tumor mass [196]. Myeloid-derived suppressor cells (MDSC) targeting compounds have been well investigated to enhance immunotherapy. MDSCs are immunosuppressive cells that suppress the immune cells in TME and also inhibit the expansion of CAR T cells [199]. It has been reported that the antitumor effects of immunotherapy using immune checkpoint antibodies, such as PD1 ab or CTLA-4 ab, were enhanced by suppressing MDSC [200,201,202]. CAR T cell activity is also enhanced by MDSC targeting agents [203,204,205,206]. Polyinosinic-polycytidylic acid diminished the suppressive activity and cell number of MDSCs in tumor-bearing mice [203]. In an NSG mouse model, all-trans retinoic acid (ATRA) eradicated monocytic MDSCs, whose expansion was induced by sarcoma and the antitumor activity of anti-GD2 CAR T cells [204]. For CD33 is overexpressed in MDSCs of cancer patients, combination therapy with gemtuzumab ozogamicin, a CD33 antibody conjugated with a cytotoxic drug from the class of calicheamicins, increased the antitumor activity of CAR T cells by depleting MDSCs [205]. Pharmacological targeting of A2aR improved CAR T cell efficacy [152,207,208]. SCH58261, an A2aR antagonist, elevated the proliferation rate and cytokine production of anti-CD19 CAR T cells [207]. As glycogen synthase kinase 3 (GSK3) is known to be inhibited by phosphorylation at the residue of Ser9 when T cells are activated [209,210], the effect of GSK3β inhibition on CAR T cell activity was investigated. The GSK3β inhibitor SB216763 increased T cell expansion and reduced exhaustion by downregulating PD1 and FasL expression in anti-IL13Rα CAR T cells [211].

### 4.2. Usage of Compounds for T Cell Ex Vivo Culture

Compounds can be used to induce ex vivo cultured T cells into undifferentiated T cells. It has been shown in preclinical and clinical studies that a high percentage of central memory CAR T cells leads to excellent antitumor efficacy (Table 2) [41,44,222,223]. Ex vivo treatment with PI3Kγ or PI3Kδ inhibitors, bromodomain and extraterminal (BET) proteins inhibitor, AKT inhibitor, or metformin has elevated the levels of CAR T cells with memory phenotype, leading to improved T cell expansion and enhanced antitumor potency [212,213,214,215,224,225,226,227]. The additions of MK2206 or AKTi, an Akt inhibitor, into CAR T cell culture increased CAR expression and induced a CD62L-expressing central memory phenotype without suppressing proliferation of CAR T cells [212,213]. Through screening of various inhibitors of epigenetic targets, JQ-1, a BET inhibitor, was found to maintain T cells with functional properties of central memory and stem cell-like T cells [214]. As a result, CAR T cells cultured in the presence of JQ-1 showed enhanced antitumor activity. T cells that were cultured in the presence of either PI3Kγ or PI3Kδ inhibitor exhibited potent antitumor activity, while those with the inhibition of both PI3Kγ and PI3Kδ were functionally impaired [215].

### 4.3. Anti-Angiogenic Agents for CAR T Cells

To improve CAR T cell efficacy in solid tumors, anti-angiogenic agents were combined with CAR T cells (Table 2) [216,217]. Anti-angiogenic agents promote leukocyte interaction, leukocyte extravasation, and tumor infiltration [228,229]. In combination with bevacizumab, a vascular endothelial growth factor (VEGF)-A antibody, anti-GD2 CAR T cells were massively infiltrated into the tumor mass to inhibit tumor growth in an orthotopic xenograft mouse model [217]. Combretastatin A-4 phosphate, a vascular disrupting agent, also significantly improved the infiltration ability of CAR T cells into solid tumors [216].

### 4.4. Safety System to Block the Side Effects of CAR T Cells

Due to severe side-effects, such as cytokine release syndrome (CRS), neurotoxicity, and on-target off-tumor effect, safety systems to block these side-effects were demanded (Table 3). The Spencer group invented a smart safety system composed of an artificial protein and a compound. This system consists of a dimerization inducer and an artificial protein, which is a caspase-9 protein tagged with mutant FK506 binding protein 12 (FKBP12) (F37V) [230]. Rimiducid, a dimer of the mutant FKBP12 ligand, induces the dimerization of caspase-9, leading to CAR T cell apoptosis [231]. This system has been proven successful in CAR T cell clinical trial [121]. Contrary to the inducible caspase 9 (iCasp9) system, there are several ‘reversible’ systems. In these systems, CAR T cell activity can be modulated ‘reversibly’ by compounds or peptides. A couple of mutant FKBP12 (mFKBP12) bearing polypeptides—scFv-CD28-mFKBP12 and mFKBP12-CD3ζ—were expressed in T cells. In this system, scFv-CD28-CD3ζ can be formed by a small molecule, rimiducid, so CAR T cell can be active only in the presence of rimiducid [48,232,233,234,235,236,237]. The proteolysis-targeting chimera (PROTAC) system can be used to modulate CAR protein levels by fusing CAR construct to bromodomain [238]. In this system, CAR T cell activity can be modulated by the PROTAC compounds of bromodomain, ARV-771 and ARV-885. In the presence of PROTAC compounds, CAR protein is degraded by the ubiquitin-proteasome pathway. Cellectis Inc. reported a CAR construct containing protease and degron domain [239]. In the absence of a protease inhibitor, asunaprevir, the degron moiety is cleaved from the CAR by protease. However, in the presence of asunaprevir, the degron domain is not cleaved from the CAR, so the CAR protein is degraded by proteolytic pathways. Cytochrome P450 family 4 subfamily B member 1 (CYP4B1) can be used as a CAR T cell suicide system. Optimized human CYP4B1, in which serine at position 427 is mutated to proline, converts prodrug 4-ipomeanol to a cytotoxic alkylating agent [240]. CAR T cells coexpressing CYP4B1 are eradicated by 4-ipomeanol. In the case of the folate receptor, we can eliminate folate receptor-overexpressing cancer cells with folic acid linked to fluorescein isothiocyanate (FITC) and anti-FITC CAR T cells [241,242]. TO-207, an mRNA 3′-end processing antagonist, blocks the side-effects of CAR T cells by inhibiting pro-inflammatory cytokine production in monocytes [243]. Dasatinib, a multi-kinase targeted inhibitor, was introduced as an efficient agent to control CAR T-cell activity [244,245]. Dasatinib downregulates CD3ζ signaling by suppressing lymphocyte-specific protein tyrosine kinase (lck). In a mouse model of CRS, dasatinib was able to block CRS by halting CAR T cell’s cytolytic activity and cytokine production.

### 4.5. Compounds Inducing Antigen Expression

Compounds can induce a synergistic effect with CAR T cells by increasing target antigen expression (Table 4) [246,247,248,249,250,251,252,253]. Inhibitors of epigenetic targets, such as histone deacetylase, histone-lysine N-methyltransferase, and DNA methyltransferase, have been shown to upregulate target antigens. Sodium valproate, a histone deacetylase inhibitor, upregulated NKG2DL expression in ovarian cancer cells to enhance the lytic activity of NKG2DL-specific CAR T cells. [246] Enhancer of zeste 2 polycomb repressive complex 2 subunit (Ezh2) inhibition upregulated GD2 expression in Ewing sarcoma [247]. The expression of CD20 on B-cell leukemia or CD38 in adult T-cell leukemia is induced by histone deacetylase inhibitor or all-trans retinoic acid [248,249]. Decitabine, a hypomethylating agent, induced strong upregulation of NY-ESO-1, a cancer/testis antigen, to enhance the efficacy of NY-ESO-1-directed immunotherapy with CAR T cells [251,254]. Several protein kinase inhibitors have been reported to upregulate specific membrane proteins. Crenolanib, an fms-related tyrosine kinase 3 (FLT3) inhibitor, increased FLT3 expression specifically in FLT3-ITD^+^ AML cell lines, and enhanced the antitumor activity of anti-FLT3 CAR T cells [255]. Sunitinib, an inhibitor of multi-tyrosine kinase, has been shown to upregulate the expression of carbonic anhydrase IX (CAIX) and enhanced the efficacy of anti-CAIX CAR T cells against renal cancer [256]. Bryostatin, a protein kinase C modulator, was shown to upregulate CD22 to improve anti-CD22 CAR T cell efficacy [250,253].

### 4.6. Usage of Vaccines to Enhance CAR T Cell Activity

Vaccines have been used to enhance CAR T cell activity in solid tumors. Vaccination targeting gp100, varicella zoster virus (VZV), Epstein–Barr virus (EBV), or Wilms tumor 1 (WT1) has been shown to enhance the antitumor activity of CAR T cells [257,258,259,260]. Injection of live recombinant vaccinia virus encoding gp100 significantly enhanced the antitumor effect of anti-Her2 CAR T cells which were manufactured with gp100-specific T cells. Vaccination with dendritic cells pulsed with antigen enhanced the antitumor effect in a xenograft model. In a clinical trial, anti-CD19 CAR T cells from EBV-specific T cells were administered to pediatric ALL patients with vaccination using EBV-transformed lymphoblastoid cell lines. The clinical results showed that the persistence of CAR T cells with vaccination was enhanced. Some groups have developed special agents to induce vaccine boosting. Ma L. et al. developed an amphiphile CAR-T ligand, which consists of an albumin-binding domain and a target peptide or protein domain [261]. This vaccine is trafficked to the lymph node due to albumin, and the target peptide is displayed on the surface of antigen presenting cells, such as dendritic cells, by which CAR T cells in lymph nodes are boosted. In a preclinical study of Reinhard, K. et al., liposomal antigen-encoding RNA, mRNA of claudin6 (CLDN6-LPX), was administered to mice, which led to the surface expression of claudin6 on DCs, and subsequent activation of CAR T cells [262].

### 4.7. Radiotherapy Combined with CAR T Cell Therapy

Recently, several groups have reported the synergistic effect of CAR T cells and radiation therapy in preclinical models [263,264,265]. In a clinical trial, radiation therapy prior to CAR T cell therapy reduced the side effects of CAR T cells and enhanced the overall response [266,267,268]. Interestingly, patients who experienced a relapse after CAR T cell therapy achieved complete remission with radiotherapy [266,269,270].

### 4.8. Compounds Used in Clinical Trials of CAR T Cells

In clinical trials, several compounds have been proven to exhibit synergistic effects with CAR T cells (Table 2) [218,219,220,221]. The clinical trial with ibrutinib, a dual inhibitor against bruton tyrosine kinase (BTK) and IL-2-inducible T cell kinase (ITK), has shown the improvement of T cell number and T cell function in CLL patients treated with ibrutinib for more than one year [271]. In addition, ibrutinib prevented CRS after CD19 CAR T cell treatment in a preclinical mouse model [272]. In patients with relapsed or refractory CLL, combination CAR T cell therapy with ibrutinib improved the probability of one-year progression-free survival and reduced the serum levels of CRS-associated cytokines [219]. A case study of a 56-year-old man with lymphoid blast phase chronic myeloid leukemia (CML) harboring the T315I mutation in the BCR-ABL fusion gene demonstrated that anti-CD19 CAR T cell therapy following dasatinib treatment induced complete remission [220]. Patients with B cell lymphoma treated with a combination of decitabine and anti-CD19 CAR T cells achieved complete remission [221].

## 5. Manufacturing Process/Ex Vivo Culture Method

The manufacturing method is critical for the success of CAR T cell therapy. The first step for CAR T cell generation is to collect peripheral blood mononuclear cells by leukapheresis [273]. In this step, the presence of monocytes suppresses the expansion of T cells [199,274]. In addition, T cells from healthy donors significantly expand and express less exhaustion markers compared to T cells from leukemic patients [162]. Moreover, the patients treated with cumulative chemotherapy cycles have low expansion potential of T cells [275]. After T cell purification, T cell activation and expansion are typically accomplished using anti-CD3/CD28 antibody-coated beads. CAR T cells expanded with anti-CD3/CD28 dynabeads demonstrate an enhanced antitumor effect in preclinical models compared to CAR T cells cultured with soluble CD3 antibody and high-dose IL-2 [276]. Several groups have used artificial antigen-presenting cells (aAPCs) expressing stimulatory receptors for T-cell expansion. Typically, irradiated K562, which is genetically modified to express OKT3 (CD3 antibody) and CD28 ligand (CD80 or CD86), has been used. CAR T cells expanded with aAPCs have shown decreased T cell exhaustion and superior antitumor activity in preclinical models compared to bead-activated CAR T cells [277,278]. The use of aAPCs platform to expand CAR T cells has been proven to be safe and successful in clinical trials [279]. IL-2 or IL7/IL15 are mostly used in CAR T cell manufacturing processes [280]. Several studies have reported that IL7/IL15 preferentially induced differentiation of CAR T cells into stem cell memory T cells (T_SCM_), while IL2 induced the differentiation of effector memory T cells (T_EM_) [39,162,281,282,283]. A high percentage of T_SCM_ by IL7/IL15 led to enhanced in vivo persistence and elevated antitumor effects in mice models. However, it is still controversial because some data demonstrated that IL7/15 is not superior to IL2 for the generation of less-differentiated subsets [284]. After two to three days of T-cell activation, the CAR gene is transduced into T cells. The most popular method to transduce the CAR gene into T cells is to use viral vectors, including lentivirus or gamma-retrovirus [285]. Due to the safety issues associated with viruses and the high cost of preparing viral particles, non-viral gene transfer methods such as sleeping beauty transposon/transposase system are employed for CAR T cell manufacturing [286,287,288,289]. Clinical trials of CAR T cell therapy with this transposon system yielded encouraging results [279,290]. Ex vivo culture time is also important for CAR T-cell function. Many reports have demonstrated that short-term culture improved antitumor effects by increasing the percentage of memory T cells and by reducing the exhaustion markers, including PD1, and TIM3 [291,292,293,294,295]. It is well known that T cells cultured ex vivo from non-responders to CAR T cell therapy exhibit high expression of PD1, TIM3, and LAG3 [296]. As most CAR T cell products are administered to patients as frozen, cryopreservation is an important step for maintaining a high quality of CAR T cells. Several studies have demonstrated that there is no significant difference in overall T cell function between cryopreserved CAR T cells and fresh CAR T cells [297,298,299]. Although the cytokine levels are slightly decreased in cryopreserved CAR T cells, antitumor function is well retained [300].

## 6. Conclusions

Although CAR T cells have shown clinical successes in hematological cancer, translating these successes to solid tumors remains a challenge. Recent studies have focused on ways to overcome the current drawbacks of CAR T cell therapy. Through the understanding of the biology of CAR T cells, many genes or signaling pathways have been revealed to enhance or decrease CAR T cell efficacy. CAR T cells genetically engineered to harness or deplete these genes or signalings have shown significantly improved antitumor effects. Combination therapy with compounds, antibodies, radiation, or vaccines has also been shown to be effective for CAR T cell therapy. However, it is still limited to using CAR T cell therapy in clinical trials for solid tumors or hematological cancers other than B cell malignancies. One of the most important issues in CAR T cell therapy against solid tumor is to uncover the tumor specific antigen. Unlike the therapy against B cell malignancies, on-target off-tumor side effects could cause a fatal problem in CAR T cell therapy for a solid tumor. One of the resolutions for this issue is to use oncolytic virus to deliver the antigen specifically to tumor cells [301]. CAR-Macrophage can be another resolution to attack solid tumor because macrophage cells are able to present the tumor antigen in an MHC-restricted manner, which boosts the adaptive immune response against tumor [302]. As the manufacturing process of autologous CAR T cell is so complicated, substantial efforts to develop allogeneic CAR T cells have been devoted. The clinical trial with allogeneic CD19 CAR T cells combined with TCRα knock out engineered T cell has shown promising result without severe graft-versus-host disease (GVHD) [303]. In addition, off-the-shelf CAR NK cell therapy has shown impressive clinical result in B-cell hematological cancer [304]. The problems of the complicated manufacturing process of CAR T cell could be solved if these cell therapies were available.

We have to admit that we still have numerous hurdles to improve CAR T cell therapy. However, we are certainly moving forward step by step to overcome these hurdles by using genetic engineering and synergistic agents. In the future, these technical advances will make it possible to conquer the cancer through CAR T cell therapy.

## Figures and Tables

**Table 1:** Proteins co-expressed or depleted in CAR T cell.

Target Proteins	Method	Effect	Reference
TERT	Expression	Escaping cell senescence; Prolonged in vivo persistence	[95]
Heparanase	Expression	Degrading heparin sulfate proteoglycan; Improved tumor infiltration	[96,97,98]
JUN	Expression	Improved IL-2 production; Escaping T cell exhaustion	[99]
PD1 or PDL1 scFv	Expression	Suppressing the immune inhibitory signaling	[100,101,102]
sPD1	Expression		[103]
Chemokine receptors (CXCR1, CXCR2, CCR2, CCR4, CCR2b)	Expression	T cell’s trafficking; Enhanced trafficking to tumor lesion	[104,105,106,107,108,109,110]
Interleukins (IL12, IL18, IL15, IL21, IL23, IL36γ, IL7)	Expression	Enhanced tumor infiltration of CAR T cell; Modulate tumor microenvironment to increase M1 macrophage; Generate T-memory stem cell	[111,112,113,114,115,116,117,118,119,120,121,122,123,124,125,126,127,128,129,130]
NR4A	shRNA	Inhibition of PD1, TIM3 expression	[131]
Tet2	shRNA	Driving T cells into central memory phenotype	[132]
PTPN2/22	shRNA	Upregulation of TCR signaling by Lck phosphorylation	[133,134,135,136]
TGFβ signaling	SD-208 (kinase inhibitor)	Increasing CAR T cell viability & suppressing PD-1 expression	[137]
	KO of TGFβ receptor	Suppressed T_reg_ conversion and CAR T cell exhaustion	[138]
	Expressing of dominant negative (DN) TGFβ receptor II	Enhanced cytokine secretion & Increased CAR T cell proliferation	[139]
Cholesterol esterification enzyme	shRNA	Elevated cholesterol level triggers the proliferation and cytotoxic activity of T cell	[140]
	inhibitor		[141]
DGK	Knockout	Reinvigorate the function of hypofunctional tumor-infiltrating immune cells	[142]
PD1	Knockout	Suppressed the immune inhibitory signaling	[143]
	PD1 decoy receptor		[144,145,146,147,148,149,150,151]
A2aR	shRNA	Increased CAR T cell efficacy	[152]

**Table 2:** Compounds enhancing CAR T cell activity.

Compounds	Function	Effect	Reference
Afatinib	Tyrosine kinase inhibitor	Increase NK cell infiltration into tumor	[192]
Lenalidomide	Immunomodulatory drug	Increase cytotoxicity, cytokine production of CAR T cell	[193]
Bortezomib	Proteasome inhibitor	Augment anti-tumor effect of CAR T cell	[195]
AMG102	HGF receptor neutralizing antibody	Increase CAR T cell trafficking into tumor	[196]
pI:pC (polyinosinic-polycytidylic acid)	Immunostimulation by interacting with toll-like receptor 3	Diminish suppressive activity and cell number of MDSC	[203]
ATRA (All-Trans Retinoic Acid)	Agonist for the retinoic acid receptors	Eradicate monocytic MDSC	[204]
Gemtuzumab ozogamicin	CD33 antibody conjugated with calicheamicins	Remove MDSC	[205]
SCH58261	A2aR antagonist	Elevate the proliferation rate and cytokine production of CAR T cell	[207]
SB216763	GSK3β inhibitor	Downregulating PD1 and FasL expression	[211]
MK2206	Akt inhibitor	Increase CAR expression; Induced CD62L expressing central memory phenotype	[212]
AKTi			[213]
JQ1	Bromodomain inhibitor	Maintain T cells with central memory and stem cell like phenotype	[214]
IPI-549	PI3Kγ inhibitor	Exhibit potent antitumor activity	[215]
CAL-101	PI3Kδ inhibitor		
Combretastatin A-4 phosphate (CA-4)	Vascular disrupting agent	Anti-angiogeneic agent; Improve the infiltration of CAR T cell into solid tumor	[216]
Bevacizumab	VEGF-a antibody		[217]
Ibrutinib	BTK/ITK inhibitor	Enhanced one-year PFS; Reduced the serum level of CRS-associated cytokines	[218,219]
Dasatinib	ABL inhibitor	Complete remission in CML patient treated with CD19 CAR T cell	[220]
Decitabine	DNA methyltransferase inhibitor	Complete remission in B cell lymphoma patients treated with CD19 CAR T cell	[221]

**Table 3:** Compounds and artificial genes used in safety system for CAR T cell therapy.

Compounds Plus Genes	Mechanism	Effect	Reference
TO-207	mRNA 3′-end processing antagonist	Block cytokine production in monocyte	[243]
Dasatinib	Multikinase targeted inhibitor	Block CRS by suppressing lymphocyte-specific protein tyrosine kinase (lck)	[244,245]
Rimiducid plus iCasp9	Dimer of mutant FKBP12 (F37V) ligand	Induce dimerization of caspase-9 leading to apoptosis	[48,231,232,233,234,235,236,237]
ARV-771 plus bromodomain containing CAR	PROTAC compound against bromodomain	Induce degradation of CAR protein leading to suppress the activity of CAR T cell	[238]
Asunaprevir plus CAR containing protease and degron	Protease inhibitor	Induce degradation of CAR protein leading to suppress the activity of CAR T cell	[239]
4-ipomeanol plus optimized CYP4B1	prodrug	Mutant CYP4B1 converts 4-ipomeanol to cytotoxic alkylating agent.	[240]

**Table 4:** Compounds upregulating specific antigens.

Compounds	Target Proteins	Reference
Sodium valproate	NKG2DL	[246]
Ezh2 inhibitor	GD2	[247]
Histone deacetylase inhibitor	CD20	[249]
ATRA	CD38	[248]
Decitabine	NY-ESO-1	[251,254]
crenolanib	FLT3	[255]
sunitinib	CAIX expression	[256]
Bryostatin	CD22	[250,253]

## References

[1] Coley W.B. (1936). The Diagnosis and Treatment of Bone Sarcoma. Glasg. Med. J..

[2] Rosenberg S.A., Spiess P., Lafreniere R. (1986). A new approach to the adoptive immunotherapy of cancer with tumor-infiltrating lymphocytes. Science.

[3] Wu R., Forget M.A., Chacon J., Bernatchez C., Haymaker C., Chen J.Q., Hwu P., Radvanyi L.G. (2012). Adoptive T-cell therapy using autologous tumor-infiltrating lymphocytes for metastatic melanoma: Current status and future outlook. Cancer J..

[4] Gross G., Waks T., Eshhar Z. (1989). Expression of immunoglobulin-T-cell receptor chimeric molecules as functional receptors with antibody-type specificity. Proc. Natl. Acad. Sci. USA.

[5] Eshhar Z., Waks T., Gross G., Schindler D.G. (1993). Specific activation and targeting of cytotoxic lymphocytes through chimeric single chains consisting of antibody-binding domains and the gamma or zeta subunits of the immunoglobulin and T-cell receptors. Proc. Natl. Acad. Sci. USA.

[6] Wang H., Wei H., Zhang R., Hou S., Li B., Qian W., Zhang D., Kou G., Dai J., Guo Y. (2009). Genetically targeted T cells eradicate established breast cancer in syngeneic mice. Clin. Cancer Res..

[7] Ahmed N., Ratnayake M., Savoldo B., Perlaky L., Dotti G., Wels W.S., Bhattacharjee M.B., Gilbertson R.J., Shine H.D., Weiss H.L. (2007). Regression of experimental medulloblastoma following transfer of HER2-specific T cells. Cancer Res..

[8] Kowolik C.M., Topp M.S., Gonzalez S., Pfeiffer T., Olivares S., Gonzalez N., Smith D.D., Forman S.J., Jensen M.C., Cooper L.J. (2006). CD28 costimulation provided through a CD19-specific chimeric antigen receptor enhances in vivo persistence and antitumor efficacy of adoptively transferred T cells. Cancer Res..

[9] Gade T.P., Hassen W., Santos E., Gunset G., Saudemont A., Gong M.C., Brentjens R., Zhong X.S., Stephan M., Stefanski J. (2005). Targeted elimination of prostate cancer by genetically directed human T lymphocytes. Cancer Res..

[10] Porter D.L., Levine B.L., Kalos M., Bagg A., June C.H. (2011). Chimeric antigen receptor-modified T cells in chronic lymphoid leukemia. N. Engl. J. Med..

[11] Brentjens R.J., Davila M.L., Riviere I., Park J., Wang X., Cowell L.G., Bartido S., Stefanski J., Taylor C., Olszewska M. (2013). CD19-targeted T cells rapidly induce molecular remissions in adults with chemotherapy-refractory acute lymphoblastic leukemia. Sci. Transl. Med..

[12] Zheng P.P., Kros J.M., Li J. (2018). Approved CAR T cell therapies: Ice bucket challenges on glaring safety risks and long-term impacts. Drug Discov. Today.

[13] Hartmann J., Schussler-Lenz M., Bondanza A., Buchholz C.J. (2017). Clinical development of CAR T cells-challenges and opportunities in translating innovative treatment concepts. EMBO Mol. Med..

[14] Jindal V., Arora E., Gupta S. (2018). Challenges and prospects of chimeric antigen receptor T cell therapy in solid tumors. Med. Oncol..

[15] Fan M., Li M., Gao L., Geng S., Wang J., Wang Y., Yan Z., Yu L. (2017). Chimeric antigen receptors for adoptive T cell therapy in acute myeloid leukemia. J. Hematol. Oncol..

[16] Weissman A.M., Baniyash M., Hou D., Samelson L.E., Burgess W.H., Klausner R.D. (1988). Molecular cloning of the zeta chain of the T cell antigen receptor. Science.

[17] Brocker T. (2000). Chimeric Fv-zeta or Fv-epsilon receptors are not sufficient to induce activation or cytokine production in peripheral T cells. Blood.

[18] Krause A., Guo H.F., Latouche J.B., Tan C., Cheung N.K., Sadelain M. (1998). Antigen-dependent CD28 signaling selectively enhances survival and proliferation in genetically modified activated human primary T lymphocytes. J. Exp. Med..

[19] Maher J., Brentjens R.J., Gunset G., Riviere I., Sadelain M. (2002). Human T-lymphocyte cytotoxicity and proliferation directed by a single chimeric TCRzeta/CD28 receptor. Nat. Biotechnol..

[20] Finney H.M., Lawson A.D., Bebbington C.R., Weir A.N. (1998). Chimeric receptors providing both primary and costimulatory signaling in T cells from a single gene product. J. Immunol..

[21] Hombach A., Wieczarkowiecz A., Marquardt T., Heuser C., Usai L., Pohl C., Seliger B., Abken H. (2001). Tumor-specific T cell activation by recombinant immunoreceptors: CD3 zeta signaling and CD28 costimulation are simultaneously required for efficient IL-2 secretion and can be integrated into one combined CD28/CD3 zeta signaling receptor molecule. J. Immunol..

[22] Wang E., Wang L.C., Tsai C.Y., Bhoj V., Gershenson Z., Moon E., Newick K., Sun J., Lo A., Baradet T. (2015). Generation of Potent T-cell Immunotherapy for Cancer Using DAP12-Based, Multichain, Chimeric Immunoreceptors. Cancer Immunol. Res..

[23] Song D.G., Ye Q., Poussin M., Harms G.M., Figini M., Powell D.J. (2012). CD27 costimulation augments the survival and antitumor activity of redirected human T cells in vivo. Blood.

[24] Song D.G., Ye Q., Carpenito C., Poussin M., Wang L.P., Ji C., Figini M., June C.H., Coukos G., Powell D.J. (2011). In vivo persistence, tumor localization, and antitumor activity of CAR-engineered T cells is enhanced by costimulatory signaling through CD137 (4-1BB). Cancer Res..

[25] Carpenito C., Milone M.C., Hassan R., Simonet J.C., Lakhal M., Suhoski M.M., Varela-Rohena A., Haines K.M., Heitjan D.F., Albelda S.M. (2009). Control of large, established tumor xenografts with genetically retargeted human T cells containing CD28 and CD137 domains. Proc. Natl. Acad. Sci. USA.

[26] Savoldo B., Ramos C.A., Liu E., Mims M.P., Keating M.J., Carrum G., Kamble R.T., Bollard C.M., Gee A.P., Mei Z. (2011). CD28 costimulation improves expansion and persistence of chimeric antigen receptor-modified T cells in lymphoma patients. J. Clin. Investig..

[27] Imai C., Mihara K., Andreansky M., Nicholson I.C., Pui C.H., Geiger T.L., Campana D. (2004). Chimeric receptors with 4-1BB signaling capacity provoke potent cytotoxicity against acute lymphoblastic leukemia. Leukemia.

[28] Shen C.J., Yang Y.X., Han E.Q., Cao N., Wang Y.F., Wang Y., Zhao Y.Y., Zhao L.M., Cui J., Gupta P. (2013). Chimeric antigen receptor containing ICOS signaling domain mediates specific and efficient antitumor effect of T cells against EGFRvIII expressing glioma. J. Hematol. Oncol..

[29] Hillerdal V., Ramachandran M., Leja J., Essand M. (2014). Systemic treatment with CAR-engineered T cells against PSCA delays subcutaneous tumor growth and prolongs survival of mice. BMC Cancer.

[30] Hombach A.A., Abken H. (2011). Costimulation by chimeric antigen receptors revisited the T cell antitumor response benefits from combined CD28-OX40 signalling. Int. J. Cancer.

[31] Wang J., Jensen M., Lin Y., Sui X., Chen E., Lindgren C.G., Till B., Raubitschek A., Forman S.J., Qian X. (2007). Optimizing adoptive polyclonal T cell immunotherapy of lymphomas, using a chimeric T cell receptor possessing CD28 and CD137 costimulatory domains. Hum. Gene Ther..

[32] Tammana S., Huang X., Wong M., Milone M.C., Ma L., Levine B.L., June C.H., Wagner J.E., Blazar B.R., Zhou X. (2010). 4-1BB and CD28 signaling plays a synergistic role in redirecting umbilical cord blood T cells against B-cell malignancies. Hum. Gene Ther..

[33] Milone M.C., Fish J.D., Carpenito C., Carroll R.G., Binder G.K., Teachey D., Samanta M., Lakhal M., Gloss B., Danet-Desnoyers G. (2009). Chimeric receptors containing CD137 signal transduction domains mediate enhanced survival of T cells and increased antileukemic efficacy in vivo. Mol. Ther..

[34] Zhong X.S., Matsushita M., Plotkin J., Riviere I., Sadelain M. (2010). Chimeric antigen receptors combining 4-1BB and CD28 signaling domains augment PI3kinase/AKT/Bcl-XL activation and CD8+ T cell-mediated tumor eradication. Mol. Ther..

[35] Ramos C.A., Rouce R., Robertson C.S., Reyna A., Narala N., Vyas G., Mehta B., Zhang H., Dakhova O., Carrum G. (2018). In Vivo Fate and Activity of Second- versus Third-Generation CD19-Specific CAR-T Cells in B Cell Non-Hodgkin’s Lymphomas. Mol. Ther..

[36] Philipson B.I., O’Connor R.S., May M.J., June C.H., Albelda S.M., Milone M.C. (2020). 4-1BB costimulation promotes CAR T cell survival through noncanonical NF-kappaB signaling. Sci. Signal..

[37] Long A.H., Haso W.M., Shern J.F., Wanhainen K.M., Murgai M., Ingaramo M., Smith J.P., Walker A.J., Kohler M.E., Venkateshwara V.R. (2015). 4-1BB costimulation ameliorates T cell exhaustion induced by tonic signaling of chimeric antigen receptors. Nat. Med..

[38] Guedan S., Posey A.D., Shaw C., Wing A., Da T., Patel P.R., McGettigan S.E., Casado-Medrano V., Kawalekar O.U., Uribe-Herranz M. (2018). Enhancing CAR T cell persistence through ICOS and 4-1BB costimulation. JCI Insight.

[39] Quintarelli C., Orlando D., Boffa I., Guercio M., Polito V.A., Petretto A., Lavarello C., Sinibaldi M., Weber G., Del Bufalo F. (2018). Choice of costimulatory domains and of cytokines determines CAR T-cell activity in neuroblastoma. Oncoimmunology.

[40] Abken H. (2016). Costimulation Engages the Gear in Driving CARs. Immunity.

[41] Li S., Tao Z., Xu Y., Liu J., An N., Wang Y., Xing H., Tian Z., Tang K., Liao X. (2018). CD33-Specific Chimeric Antigen Receptor T Cells with Different Co-Stimulators Showed Potent Anti-Leukemia Efficacy and Different Phenotype. Hum. Gene Ther..

[42] Ying Z., He T., Wang X., Zheng W., Lin N., Tu M., Xie Y., Ping L., Zhang C., Liu W. (2019). Parallel Comparison of 4-1BB or CD28 Co-stimulated CD19-Targeted CAR-T Cells for B Cell Non-Hodgkin’s Lymphoma. Mol. Ther. Oncolytics.

[43] Zhang L.N., Song Y., Liu D. (2018). CD19 CAR-T cell therapy for relapsed/refractory acute lymphoblastic leukemia: Factors affecting toxicities and long-term efficacies. J. Hematol. Oncol..

[44] Wang X., Popplewell L.L., Wagner J.R., Naranjo A., Blanchard M.S., Mott M.R., Norris A.P., Wong C.W., Urak R.Z., Chang W.C. (2016). Phase 1 studies of central memory-derived CD19 CAR T-cell therapy following autologous HSCT in patients with B-cell NHL. Blood.

[45] Drent E., Poels R., Ruiter R., van de Donk N., Zweegman S., Yuan H., de Bruijn J., Sadelain M., Lokhorst H.M., Groen R.W.J. (2019). Combined CD28 and 4-1BB Costimulation Potentiates Affinity-tuned Chimeric Antigen Receptor-engineered T Cells. Clin. Cancer Res..

[46] Hu W., Huang X., Huang X., Chen W., Hao L., Chen Z. (2019). Chimeric antigen receptor modified T cell (CAR-T) co-expressed with ICOSL-41BB promote CAR-T proliferation and tumor rejection. Biomed. Pharmacother..

[47] Nunoya J.I., Masuda M., Ye C., Su L. (2019). Chimeric Antigen Receptor T Cell Bearing Herpes Virus Entry Mediator Co-stimulatory Signal Domain Exhibits High Functional Potency. Mol. Ther. Oncolytics.

[48] Mata M., Gerken C., Nguyen P., Krenciute G., Spencer D.M., Gottschalk S. (2017). Inducible Activation of MyD88 and CD40 in CAR T Cells Results in Controllable and Potent Antitumor Activity in Preclinical Solid Tumor Models. Cancer Discov..

[49] Wang X., Jasinski D.L., Medina J.L., Spencer D.M., Foster A.E., Bayle J.H. (2020). Inducible MyD88/CD40 synergizes with IL-15 to enhance antitumor efficacy of CAR-NK cells. Blood Adv..

[50] Weng J., Lai P., Qin L., Lai Y., Jiang Z., Luo C., Huang X., Wu S., Shao D., Deng C. (2018). A novel generation 1928zT2 CAR T cells induce remission in extramedullary relapse of acute lymphoblastic leukemia. J. Hematol. Oncol..

[51] George P., Dasyam N., Giunti G., Mester B., Bauer E., Andrews B., Perera T., Ostapowicz T., Frampton C., Li P. (2020). Third-generation anti-CD19 chimeric antigen receptor T-cells incorporating a TLR2 domain for relapsed or refractory B-cell lymphoma: A phase I clinical trial protocol (ENABLE). BMJ Open.

[52] Lai Y., Weng J., Wei X., Qin L., Lai P., Zhao R., Jiang Z., Li B., Lin S., Wang S. (2018). Toll-like receptor 2 costimulation potentiates the antitumor efficacy of CAR T Cells. Leukemia.

[53] Kagoya Y., Tanaka S., Guo T., Anczurowski M., Wang C.H., Saso K., Butler M.O., Minden M.D., Hirano N. (2018). A novel chimeric antigen receptor containing a JAK-STAT signaling domain mediates superior antitumor effects. Nat. Med..

[54] Nair S., Wang J.B., Tsao S.T., Liu Y., Zhu W., Slayton W.B., Moreb J.S., Dong L., Chang L.J. (2019). Functional Improvement of Chimeric Antigen Receptor Through Intrinsic Interleukin-15Ralpha Signaling. Curr. Gene Ther..

[55] Alabanza L., Pegues M., Geldres C., Shi V., Wiltzius J.J.W., Sievers S.A., Yang S., Kochenderfer J.N. (2017). Function of Novel Anti-CD19 Chimeric Antigen Receptors with Human Variable Regions Is Affected by Hinge and Transmembrane Domains. Mol. Ther..

[56] Ying Z., Huang X.F., Xiang X., Liu Y., Kang X., Song Y., Guo X., Liu H., Ding N., Zhang T. (2019). A safe and potent anti-CD19 CAR T cell therapy. Nat. Med..

[57] Fujiwara K., Tsunei A., Kusabuka H., Ogaki E., Tachibana M., Okada N. (2020). Hinge and Transmembrane Domains of Chimeric Antigen Receptor Regulate Receptor Expression and Signaling Threshold. Cells.

[58] Jonnalagadda M., Mardiros A., Urak R., Wang X., Hoffman L.J., Bernanke A., Chang W.C., Bretzlaff W., Starr R., Priceman S. (2015). Chimeric antigen receptors with mutated IgG4 Fc spacer avoid fc receptor binding and improve T cell persistence and antitumor efficacy. Mol. Ther..

[59] Hudecek M., Sommermeyer D., Kosasih P.L., Silva-Benedict A., Liu L., Rader C., Jensen M.C., Riddell S.R. (2015). The nonsignaling extracellular spacer domain of chimeric antigen receptors is decisive for in vivo antitumor activity. Cancer Immunol. Res..

[60] Wan Z., Shao X., Ji X., Dong L., Wei J., Xiong Z., Liu W., Qi H. (2020). Transmembrane domain-mediated Lck association underlies bystander and costimulatory ICOS signaling. Cell. Mol. Immunol..

[61] Youngblood B., Davis C.W., Ahmed R. (2010). Making memories that last a lifetime: Heritable functions of self-renewing memory CD8 T cells. Int. Immunol..

[62] Feucht J., Sun J., Eyquem J., Ho Y.J., Zhao Z., Leibold J., Dobrin A., Cabriolu A., Hamieh M., Sadelain M. (2019). Calibration of CAR activation potential directs alternative T cell fates and therapeutic potency. Nat. Med..

[63] Guedan S., Madar A., Casado-Medrano V., Shaw C., Wing A., Liu F., Young R.M., June C.H., Posey A.D. (2020). Single residue in CD28-costimulated CAR-T cells limits long-term persistence and antitumor durability. J. Clin. Investig..

[64] Wijewarnasuriya D., Bebernitz C., Lopez A.V., Rafiq S., Brentjens R.J. (2020). Excessive Costimulation Leads to Dysfunction of Adoptively Transferred T Cells. Cancer Immunol. Res..

[65] Lamers C.H., Willemsen R., van Elzakker P., van Steenbergen-Langeveld S., Broertjes M., Oosterwijk-Wakka J., Oosterwijk E., Sleijfer S., Debets R., Gratama J.W. (2011). Immune responses to transgene and retroviral vector in patients treated with ex vivo-engineered T cells. Blood.

[66] Turtle C.J., Hanafi L.A., Berger C., Gooley T.A., Cherian S., Hudecek M., Sommermeyer D., Melville K., Pender B., Budiarto T.M. (2016). CD19 CAR-T cells of defined CD4+:CD8+ composition in adult B cell ALL patients. J. Clin. Investig..

[67] Maus M.V., Haas A.R., Beatty G.L., Albelda S.M., Levine B.L., Liu X., Zhao Y., Kalos M., June C.H. (2013). T cells expressing chimeric antigen receptors can cause anaphylaxis in humans. Cancer Immunol. Res..

[68] Zheng L., Ren L., Kouhi A., Khawli L.A., Hu P., Kaslow H.R., Epstein A.L. (2020). A Humanized Lym-1 CAR with Novel DAP10/DAP12 Signaling Domains Demonstrates Reduced Tonic Signaling and Increased Antitumor Activity in B-Cell Lymphoma Models. Clin. Cancer Res..

[69] Qian L., Li D., Ma L., He T., Qi F., Shen J., Lu X.A. (2016). The novel anti-CD19 chimeric antigen receptors with humanized scFv (single-chain variable fragment) trigger leukemia cell killing. Cell. Immunol..

[70] Smith E.L., Staehr M., Masakayan R., Tatake I.J., Purdon T.J., Wang X., Wang P., Liu H., Xu Y., Garrett-Thomson S.C. (2018). Development and Evaluation of an Optimal Human Single-Chain Variable Fragment-Derived BCMA-Targeted CAR T Cell Vector. Mol. Ther..

[71] Perez-Amill L., Sune G., Antonana-Vildosola A., Castella M., Najjar A., Bonet J., Fernandez-Fuentes N., Inoges S., Lopez A., Bueno C. (2020). Preclinical development of a humanized chimeric antigen receptor against B cell maturation antigen for multiple myeloma. Haematologica.

[72] Festag M.M., Festag J., Frassle S.P., Asen T., Sacherl J., Schreiber S., Muck-Hausl M.A., Busch D.H., Wisskirchen K., Protzer U. (2019). Evaluation of a Fully Human, Hepatitis B Virus-Specific Chimeric Antigen Receptor in an Immunocompetent Mouse Model. Mol. Ther..

[73] Lam N., Trinklein N.D., Buelow B., Patterson G.H., Ojha N., Kochenderfer J.N. (2020). Anti-BCMA chimeric antigen receptors with fully human heavy-chain-only antigen recognition domains. Nat. Commun..

[74] Mirzaei H.R., Jamali A., Jafarzadeh L., Masoumi E., Alishah K., Fallah Mehrjardi K., Emami S.A.H., Noorbakhsh F., Till B.G., Hadjati J. (2019). Construction and functional characterization of a fully human anti-CD19 chimeric antigen receptor (huCAR)-expressing primary human T cells. J. Cell. Physiol..

[75] Brudno J.N., Lam N., Vanasse D., Shen Y.W., Rose J.J., Rossi J., Xue A., Bot A., Scholler N., Mikkilineni L. (2020). Safety and feasibility of anti-CD19 CAR T cells with fully human binding domains in patients with B-cell lymphoma. Nat. Med..

[76] Yang F., Zhang J., Zhang X., Tian M., Wang J., Kang L., Qiu H., Wu D. (2019). Delayed remission following sequential infusion of humanized CD19- and CD22-modified CAR-T cells in a patient with relapsed/refractory acute lymphoblastic leukemia and prior exposure to murine-derived CD19-directed CAR-T cells. OncoTargets Ther..

[77] Wang J., Mou N., Yang Z., Li Q., Jiang Y., Meng J., Liu X., Deng Q. (2020). Efficacy and safety of humanized anti-CD19-CAR-T therapy following intensive lymphodepleting chemotherapy for refractory/relapsed B acute lymphoblastic leukaemia. Br. J. Haematol..

[78] Heng G., Jia J., Li S., Fu G., Wang M., Qin D., Li Y., Pei L., Tian X., Zhang J. (2020). Sustained Therapeutic Efficacy of Humanized Anti-CD19 Chimeric Antigen Receptor T Cells in Relapsed/Refractory Acute Lymphoblastic Leukemia. Clin. Cancer Res..

[79] Jarboe J.S., Johnson K.R., Choi Y., Lonser R.R., Park J.K. (2007). Expression of interleukin-13 receptor alpha2 in glioblastoma multiforme: Implications for targeted therapies. Cancer Res..

[80] Debinski W., Gibo D.M., Hulet S.W., Connor J.R., Gillespie G.Y. (1999). Receptor for interleukin 13 is a marker and therapeutic target for human high-grade gliomas. Clin. Cancer Res..

[81] Hegde M., Mukherjee M., Grada Z., Pignata A., Landi D., Navai S.A., Wakefield A., Fousek K., Bielamowicz K., Chow K.K. (2019). Tandem CAR T cells targeting HER2 and IL13Ralpha2 mitigate tumor antigen escape. J. Clin. Investig..

[82] Brown C.E., Aguilar B., Starr R., Yang X., Chang W.C., Weng L., Chang B., Sarkissian A., Brito A., Sanchez J.F. (2018). Optimization of IL13Ralpha2-Targeted Chimeric Antigen Receptor T Cells for Improved Anti-tumor Efficacy against Glioblastoma. Mol. Ther..

[83] Brown C.E., Badie B., Barish M.E., Weng L., Ostberg J.R., Chang W.C., Naranjo A., Starr R., Wagner J., Wright C. (2015). Bioactivity and Safety of IL13Ralpha2-Redirected Chimeric Antigen Receptor CD8+ T Cells in Patients with Recurrent Glioblastoma. Clin. Cancer Res..

[84] Brown C.E., Alizadeh D., Starr R., Weng L., Wagner J.R., Naranjo A., Ostberg J.R., Blanchard M.S., Kilpatrick J., Simpson J. (2016). Regression of Glioblastoma after Chimeric Antigen Receptor T-Cell Therapy. N. Engl. J. Med..

[85] Nakazawa Y., Matsuda K., Kurata T., Sueki A., Tanaka M., Sakashita K., Imai C., Wilson M.H., Koike K. (2016). Anti-proliferative effects of T cells expressing a ligand-based chimeric antigen receptor against CD116 on CD34(+) cells of juvenile myelomonocytic leukemia. J. Hematol. Oncol..

[86] Shaffer D.R., Savoldo B., Yi Z., Chow K.K., Kakarla S., Spencer D.M., Dotti G., Wu M.F., Liu H., Kenney S. (2011). T cells redirected against CD70 for the immunotherapy of CD70-positive malignancies. Blood.

[87] Park Y.P., Jin L., Bennett K.B., Wang D., Fredenburg K.M., Tseng J.E., Chang L.J., Huang J., Chan E.K.L. (2018). CD70 as a target for chimeric antigen receptor T cells in head and neck squamous cell carcinoma. Oral Oncol..

[88] Wang Q.J., Yu Z., Hanada K.I., Patel K., Kleiner D., Restifo N.P., Yang J.C. (2017). Preclinical Evaluation of Chimeric Antigen Receptors Targeting CD70-Expressing Cancers. Clin. Cancer Res..

[89] Huang G., Yu L., Cooper L.J., Hollomon M., Huls H., Kleinerman E.S. (2012). Genetically modified T cells targeting interleukin-11 receptor alpha-chain kill human osteosarcoma cells and induce the regression of established osteosarcoma lung metastases. Cancer Res..

[90] Wang D., Starr R., Chang W.C., Aguilar B., Alizadeh D., Wright S.L., Yang X., Brito A., Sarkissian A., Ostberg J.R. (2020). Chlorotoxin-directed CAR T cells for specific and effective targeting of glioblastoma. Sci. Transl. Med..

[91] Pluckthun A. (2015). Designed ankyrin repeat proteins (DARPins): Binding proteins for research, diagnostics, and therapy. Annu. Rev. Pharmacol. Toxicol..

[92] Hammill J.A., VanSeggelen H., Helsen C.W., Denisova G.F., Evelegh C., Tantalo D.G., Bassett J.D., Bramson J.L. (2015). Designed ankyrin repeat proteins are effective targeting elements for chimeric antigen receptors. J. Immunother. Cancer.

[93] Siegler E., Li S., Kim Y.J., Wang P. (2017). Designed Ankyrin Repeat Proteins as Her2 Targeting Domains in Chimeric Antigen Receptor-Engineered T Cells. Hum. Gene Ther..

[94] Balakrishnan A., Rajan A., Salter A.I., Kosasih P.L., Wu Q., Voutsinas J., Jensen M.C., Pluckthun A., Riddell S.R. (2019). Multispecific Targeting with Synthetic Ankyrin Repeat Motif Chimeric Antigen Receptors. Clin. Cancer Res..

[95] Bai Y., Kan S., Zhou S., Wang Y., Xu J., Cooke J.P., Wen J., Deng H. (2015). Enhancement of the in vivo persistence and antitumor efficacy of CD19 chimeric antigen receptor T cells through the delivery of modified TERT mRNA. Cell Discov..

[96] Vlodavsky I., Ilan N., Naggi A., Casu B. (2007). Heparanase: Structure, biological functions, and inhibition by heparin-derived mimetics of heparan sulfate. Curr. Pharm. Des..

[97] de Mestre A.M., Staykova M.A., Hornby J.R., Willenborg D.O., Hulett M.D. (2007). Expression of the heparan sulfate-degrading enzyme heparanase is induced in infiltrating CD4(+) T cells in experimental autoimmune encephalomyelitis and regulated at the level of transcription by early growth response gene. J. Leukoc. Biol..

[98] Caruana I., Savoldo B., Hoyos V., Weber G., Liu H., Kim E.S., Ittmann M.M., Marchetti D., Dotti G. (2015). Heparanase promotes tumor infiltration and antitumor activity of CAR-redirected T lymphocytes. Nat. Med..

[99] Lynn R.C., Weber E.W., Sotillo E., Gennert D., Xu P., Good Z., Anbunathan H., Lattin J., Jones R., Tieu V. (2019). c-Jun overexpression in CAR T cells induces exhaustion resistance. Nature.

[100] Li S., Siriwon N., Zhang X., Yang S., Jin T., He F., Kim Y.J., Mac J., Lu Z., Wang S. (2017). Enhanced Cancer Immunotherapy by Chimeric Antigen Receptor-Modified T Cells Engineered to Secrete Checkpoint Inhibitors. Clin. Cancer Res..

[101] Rafiq S., Yeku O.O., Jackson H.J., Purdon T.J., van Leeuwen D.G., Drakes D.J., Song M., Miele M.M., Li Z., Wang P. (2018). Targeted delivery of a PD-1-blocking scFv by CAR-T cells enhances anti-tumor efficacy in vivo. Nat. Biotechnol..

[102] Suarez E.R., Chang de K., Sun J., Sui J., Freeman G.J., Signoretti S., Zhu Q., Marasco W.A. (2016). Chimeric antigen receptor T cells secreting anti-PD-L1 antibodies more effectively regress renal cell carcinoma in a humanized mouse model. Oncotarget.

[103] Pan Z., Di S., Shi B., Jiang H., Shi Z., Liu Y., Wang Y., Luo H., Yu M., Wu X. (2018). Increased antitumor activities of glypican-3-specific chimeric antigen receptor-modified T cells by coexpression of a soluble PD1-CH3 fusion protein. Cancer Immunol. Immunother..

[104] Ng Y.Y., Tay J.C.K., Wang S. (2020). CXCR1 Expression to Improve Anti-Cancer Efficacy of Intravenously Injected CAR-NK Cells in Mice with Peritoneal Xenografts. Mol. Ther. Oncolytics.

[105] Whilding L.M., Halim L., Draper B., Parente-Pereira A.C., Zabinski T., Davies D.M., Maher J. (2019). CAR T-Cells Targeting the Integrin alphavbeta6 and Co-Expressing the Chemokine Receptor CXCR2 Demonstrate Enhanced Homing and Efficacy against Several Solid Malignancies. Cancers.

[106] Liu G., Rui W., Zheng H., Huang D., Yu F., Zhang Y., Dong J., Zhao X., Lin X. (2020). CXCR2-modified CAR-T cells have enhanced trafficking ability that improves treatment of hepatocellular carcinoma. Eur. J. Immunol..

[107] Moon E.K., Carpenito C., Sun J., Wang L.C., Kapoor V., Predina J., Powell D.J., Riley J.L., June C.H., Albelda S.M. (2011). Expression of a functional CCR2 receptor enhances tumor localization and tumor eradication by retargeted human T cells expressing a mesothelin-specific chimeric antibody receptor. Clin. Cancer Res..

[108] Di Stasi A., De Angelis B., Rooney C.M., Zhang L., Mahendravada A., Foster A.E., Heslop H.E., Brenner M.K., Dotti G., Savoldo B. (2009). T lymphocytes coexpressing CCR4 and a chimeric antigen receptor targeting CD30 have improved homing and antitumor activity in a Hodgkin tumor model. Blood.

[109] Craddock J.A., Lu A., Bear A., Pule M., Brenner M.K., Rooney C.M., Foster A.E. (2010). Enhanced tumor trafficking of GD2 chimeric antigen receptor T cells by expression of the chemokine receptor CCR2b. J. Immunother..

[110] Jin L., Tao H., Karachi A., Long Y., Hou A.Y., Na M., Dyson K.A., Grippin A.J., Deleyrolle L.P., Zhang W. (2019). CXCR1- or CXCR2-modified CAR T cells co-opt IL-8 for maximal antitumor efficacy in solid tumors. Nat. Commun..

[111] Yeku O.O., Purdon T.J., Koneru M., Spriggs D., Brentjens R.J. (2017). Armored CAR T cells enhance antitumor efficacy and overcome the tumor microenvironment. Sci. Rep..

[112] Koneru M., Purdon T.J., Spriggs D., Koneru S., Brentjens R.J. (2015). IL-12 secreting tumor-targeted chimeric antigen receptor T cells eradicate ovarian tumors in vivo. Oncoimmunology.

[113] Chmielewski M., Kopecky C., Hombach A.A., Abken H. (2011). IL-12 release by engineered T cells expressing chimeric antigen receptors can effectively Muster an antigen-independent macrophage response on tumor cells that have shut down tumor antigen expression. Cancer Res..

[114] Pegram H.J., Lee J.C., Hayman E.G., Imperato G.H., Tedder T.F., Sadelain M., Brentjens R.J. (2012). Tumor-targeted T cells modified to secrete IL-12 eradicate systemic tumors without need for prior conditioning. Blood.

[115] Chinnasamy D., Yu Z., Kerkar S.P., Zhang L., Morgan R.A., Restifo N.P., Rosenberg S.A. (2012). Local delivery of interleukin-12 using T cells targeting VEGF receptor-2 eradicates multiple vascularized tumors in mice. Clin. Cancer Res..

[116] Luo H., Wu X., Sun R., Su J., Wang Y., Dong Y., Shi B., Sun Y., Jiang H., Li Z. (2019). Target-Dependent Expression of IL12 by synNotch Receptor-Engineered NK92 Cells Increases the Antitumor Activities of CAR-T Cells. Front. Oncol..

[117] Kueberuwa G., Kalaitsidou M., Cheadle E., Hawkins R.E., Gilham D.E. (2018). CD19 CAR T Cells Expressing IL-12 Eradicate Lymphoma in Fully Lymphoreplete Mice through Induction of Host Immunity. Mol. Ther. Oncolytics.

[118] Avanzi M.P., Yeku O., Li X., Wijewarnasuriya D.P., van Leeuwen D.G., Cheung K., Park H., Purdon T.J., Daniyan A.F., Spitzer M.H. (2018). Engineered Tumor-Targeted T Cells Mediate Enhanced Anti-Tumor Efficacy Both Directly and through Activation of the Endogenous Immune System. Cell Rep..

[119] Hu B., Ren J., Luo Y., Keith B., Young R.M., Scholler J., Zhao Y., June C.H. (2017). Augmentation of Antitumor Immunity by Human and Mouse CAR T Cells Secreting IL-18. Cell Rep..

[120] Chmielewski M., Abken H. (2017). CAR T Cells Releasing IL-18 Convert to T-Bet(high) FoxO1(low) Effectors that Exhibit Augmented Activity against Advanced Solid Tumors. Cell Rep..

[121] Hoyos V., Savoldo B., Quintarelli C., Mahendravada A., Zhang M., Vera J., Heslop H.E., Rooney C.M., Brenner M.K., Dotti G. (2010). Engineering CD19-specific T lymphocytes with interleukin-15 and a suicide gene to enhance their anti-lymphoma/leukemia effects and safety. Leukemia.

[122] Hurton L.V., Singh H., Najjar A.M., Switzer K.C., Mi T., Maiti S., Olivares S., Rabinovich B., Huls H., Forget M.A. (2016). Tethered IL-15 augments antitumor activity and promotes a stem-cell memory subset in tumor-specific T cells. Proc. Natl. Acad. Sci. USA.

[123] Krenciute G., Prinzing B.L., Yi Z., Wu M.F., Liu H., Dotti G., Balyasnikova I.V., Gottschalk S. (2017). Transgenic Expression of IL15 Improves Antiglioma Activity of IL13Ralpha2-CAR T Cells but Results in Antigen Loss Variants. Cancer Immunol. Res..

[124] Batra S.A., Rathi P., Guo L., Courtney A.N., Fleurence J., Balzeau J., Shaik R.S., Nguyen T.P., Wu M.F., Bulsara S. (2020). Glypican-3-Specific CAR T Cells Coexpressing IL15 and IL21 Have Superior Expansion and Antitumor Activity against Hepatocellular Carcinoma. Cancer Immunol. Res..

[125] Ma X., Shou P., Smith C., Chen Y., Du H., Sun C., Porterfield Kren N., Michaud D., Ahn S., Vincent B. (2020). Interleukin-23 engineering improves CAR T cell function in solid tumors. Nat. Biotechnol..

[126] Li X., Daniyan A.F., Lopez A.V., Purdon T.J., Brentjens R.J. (2021). Cytokine IL-36gamma improves CAR T-cell functionality and induces endogenous antitumor response. Leukemia.

[127] Adachi K., Kano Y., Nagai T., Okuyama N., Sakoda Y., Tamada K. (2018). IL-7 and CCL19 expression in CAR-T cells improves immune cell infiltration and CAR-T cell survival in the tumor. Nat. Biotechnol..

[128] Caughron B., Yang Y., Young M.R.I. (2018). Role of IL-23 signaling in the progression of premalignant oral lesions to cancer. PLoS ONE.

[129] Nie W., Yu T., Sang Y., Gao X. (2017). Tumor-promoting effect of IL-23 in mammary cancer mediated by infiltration of M2 macrophages and neutrophils in tumor microenvironment. Biochem. Biophys. Res. Commun..

[130] Wang D., Shao Y., Zhang X., Lu G., Liu B. (2020). IL-23 and PSMA-targeted duo-CAR T cells in Prostate Cancer Eradication in a preclinical model. J. Transl. Med..

[131] Chen J., Lopez-Moyado I.F., Seo H., Lio C.J., Hempleman L.J., Sekiya T., Yoshimura A., Scott-Browne J.P., Rao A. (2019). NR4A transcription factors limit CAR T cell function in solid tumours. Nature.

[132] Fraietta J.A., Nobles C.L., Sammons M.A., Lundh S., Carty S.A., Reich T.J., Cogdill A.P., Morrissette J.J.D., DeNizio J.E., Reddy S. (2018). Disruption of TET2 promotes the therapeutic efficacy of CD19-targeted T cells. Nature.

[133] Wiede F., Shields B.J., Chew S.H., Kyparissoudis K., van Vliet C., Galic S., Tremblay M.L., Russell S.M., Godfrey D.I., Tiganis T. (2011). T cell protein tyrosine phosphatase attenuates T cell signaling to maintain tolerance in mice. J. Clin. Investig..

[134] Brownlie R.J., Zamoyska R., Salmond R.J. (2018). Regulation of autoimmune and anti-tumour T-cell responses by PTPN22. Immunology.

[135] Brownlie R.J., Wright D., Zamoyska R., Salmond R.J. (2019). Deletion of PTPN22 improves effector and memory CD8+ T cell responses to tumors. JCI Insight.

[136] Wiede F., Lu K.H., Du X., Liang S., Hochheiser K., Dodd G.T., Goh P.K., Kearney C., Meyran D., Beavis P.A. (2020). PTPN2 phosphatase deletion in T cells promotes anti-tumour immunity and CAR T-cell efficacy in solid tumours. EMBO J..

[137] Stuber T., Monjezi R., Wallstabe L., Kuhnemundt J., Nietzer S.L., Dandekar G., Wockel A., Einsele H., Wischhusen J., Hudecek M. (2020). Inhibition of TGF-beta-receptor signaling augments the antitumor function of ROR1-specific CAR T-cells against triple-negative breast cancer. J. Immunother. Cancer.

[138] Tang N., Cheng C., Zhang X., Qiao M., Li N., Mu W., Wei X.F., Han W., Wang H. (2020). TGF-beta inhibition via CRISPR promotes the long-term efficacy of CAR T cells against solid tumors. JCI Insight.

[139] Kloss C.C., Lee J., Zhang A., Chen F., Melenhorst J.J., Lacey S.F., Maus M.V., Fraietta J.A., Zhao Y., June C.H. (2018). Dominant-Negative TGF-beta Receptor Enhances PSMA-Targeted Human CAR T Cell Proliferation And Augments Prostate Cancer Eradication. Mol. Ther..

[140] Zhao L., Liu Y., Zhao F., Jin Y., Feng J., Geng R., Sun J., Kang L., Yu L., Wei Y. (2020). Inhibition of Cholesterol Esterification Enzyme Enhances the Potency of Human Chimeric Antigen Receptor T Cells against Pancreatic Carcinoma. Mol. Ther. Oncolytics.

[141] Zhao L., Li J., Liu Y., Kang L., Chen H., Jin Y., Zhao F., Feng J., Fang C., Zhu B. (2018). Cholesterol Esterification Enzyme Inhibition Enhances Antitumor Effects of Human Chimeric Antigen Receptors Modified T Cells. J. Immunother..

[142] Jung I.Y., Kim Y.Y., Yu H.S., Lee M., Kim S., Lee J. (2018). CRISPR/Cas9-Mediated Knockout of DGK Improves Antitumor Activities of Human T Cells. Cancer Res..

[143] Liu X., Zhang Y., Cheng C., Cheng A.W., Zhang X., Li N., Xia C., Wei X., Liu X., Wang H. (2017). CRISPR-Cas9-mediated multiplex gene editing in CAR-T cells. Cell Res..

[144] Kobold S., Grassmann S., Chaloupka M., Lampert C., Wenk S., Kraus F., Rapp M., Duwell P., Zeng Y., Schmollinger J.C. (2015). Impact of a New Fusion Receptor on PD-1-Mediated Immunosuppression in Adoptive T Cell Therapy. J. Natl. Cancer Inst..

[145] Liu X., Ranganathan R., Jiang S., Fang C., Sun J., Kim S., Newick K., Lo A., June C.H., Zhao Y. (2016). A Chimeric Switch-Receptor Targeting PD1 Augments the Efficacy of Second-Generation CAR T Cells in Advanced Solid Tumors. Cancer Res..

[146] Ankri C., Shamalov K., Horovitz-Fried M., Mauer S., Cohen C.J. (2013). Human T cells engineered to express a programmed death 1/28 costimulatory retargeting molecule display enhanced antitumor activity. J. Immunol..

[147] Prosser M.E., Brown C.E., Shami A.F., Forman S.J., Jensen M.C. (2012). Tumor PD-L1 co-stimulates primary human CD8(+) cytotoxic T cells modified to express a PD1:CD28 chimeric receptor. Mol. Immunol..

[148] Guo C., Wang X., Zhang H., Zhi L., Lv T., Li M., Lu C., Zhu W. (2019). Structure-based rational design of a novel chimeric PD1-NKG2D receptor for natural killer cells. Mol. Immunol..

[149] Lu C., Guo C., Chen H., Zhang H., Zhi L., Lv T., Li M., Niu Z., Lu P., Zhu W. (2020). A novel chimeric PD1-NKG2D-41BB receptor enhances antitumor activity of NK92 cells against human lung cancer H1299 cells by triggering pyroptosis. Mol. Immunol..

[150] Kintz H., Nylen E., Barber A. (2020). Inclusion of Dap10 or 4-1BB costimulation domains in the chPD1 receptor enhances anti-tumor efficacy of T cells in murine models of lymphoma and melanoma. Cell. Immunol..

[151] Parriott G., Deal K., Crean S., Richardson E., Nylen E., Barber A. (2020). T-cells expressing a chimeric-PD1-Dap10-CD3zeta receptor reduce tumour burden in multiple murine syngeneic models of solid cancer. Immunology.

[152] Beavis P.A., Henderson M.A., Giuffrida L., Mills J.K., Sek K., Cross R.S., Davenport A.J., John L.B., Mardiana S., Slaney C.Y. (2017). Targeting the adenosine 2A receptor enhances chimeric antigen receptor T cell efficacy. J. Clin. Investig..

[153] Gagnon E., Schubert D.A., Gordo S., Chu H.H., Wucherpfennig K.W. (2012). Local changes in lipid environment of TCR microclusters regulate membrane binding by the CD3epsilon cytoplasmic domain. J. Exp. Med..

[154] Yang W., Bai Y., Xiong Y., Zhang J., Chen S., Zheng X., Meng X., Li L., Wang J., Xu C. (2016). Potentiating the antitumour response of CD8(+) T cells by modulating cholesterol metabolism. Nature.

[155] Ohta A., Ohta A., Madasu M., Kini R., Subramanian M., Goel N., Sitkovsky M. (2009). A2A adenosine receptor may allow expansion of T cells lacking effector functions in extracellular adenosine-rich microenvironments. J. Immunol..

[156] Ohta A., Gorelik E., Prasad S.J., Ronchese F., Lukashev D., Wong M.K., Huang X., Caldwell S., Liu K., Smith P. (2006). A2A adenosine receptor protects tumors from antitumor T cells. Proc. Natl. Acad. Sci. USA.

[157] Li Y., Xiao F., Zhang A., Zhang D., Nie W., Xu T., Han B., Seth P., Wang H., Yang Y. (2020). Oncolytic adenovirus targeting TGF-beta enhances anti-tumor responses of mesothelin-targeted chimeric antigen receptor T cell therapy against breast cancer. Cell. Immunol..

[158] Eyquem J., Mansilla-Soto J., Giavridis T., van der Stegen S.J., Hamieh M., Cunanan K.M., Odak A., Gonen M., Sadelain M. (2017). Targeting a CAR to the TRAC locus with CRISPR/Cas9 enhances tumour rejection. Nature.

[159] Prinz P.U., Mendler A.N., Masouris I., Durner L., Oberneder R., Noessner E. (2012). High DGK-alpha and disabled MAPK pathways cause dysfunction of human tumor-infiltrating CD8+ T cells that is reversible by pharmacologic intervention. J. Immunol..

[160] Zhang Y., Zhang X., Cheng C., Mu W., Liu X., Li N., Wei X., Liu X., Xia C., Wang H. (2017). CRISPR-Cas9 mediated LAG-3 disruption in CAR-T cells. Front. Med..

[161] Moon E.K., Wang L.C., Dolfi D.V., Wilson C.B., Ranganathan R., Sun J., Kapoor V., Scholler J., Pure E., Milone M.C. (2014). Multifactorial T-cell hypofunction that is reversible can limit the efficacy of chimeric antigen receptor-transduced human T cells in solid tumors. Clin. Cancer Res..

[162] Hoffmann J.M., Schubert M.L., Wang L., Huckelhoven A., Sellner L., Stock S., Schmitt A., Kleist C., Gern U., Loskog A. (2017). Differences in Expansion Potential of Naive Chimeric Antigen Receptor T Cells from Healthy Donors and Untreated Chronic Lymphocytic Leukemia Patients. Front. Immunol..

[163] Fraietta J.A., Lacey S.F., Orlando E.J., Pruteanu-Malinici I., Gohil M., Lundh S., Boesteanu A.C., Wang Y., O’Connor R.S., Hwang W.T. (2018). Determinants of response and resistance to CD19 chimeric antigen receptor (CAR) T cell therapy of chronic lymphocytic leukemia. Nat. Med..

[164] Fedorov V.D., Themeli M., Sadelain M. (2013). PD-1- and CTLA-4-based inhibitory chimeric antigen receptors (iCARs) divert off-target immunotherapy responses. Sci. Transl. Med..

[165] Xin Yu J., Hodge J.P., Oliva C., Neftelinov S.T., Hubbard-Lucey V.M., Tang J. (2020). Trends in clinical development for PD-1/PD-L1 inhibitors. Nat. Rev. Drug Discov..

[166] Gulati P., Ruhl J., Kannan A., Pircher M., Schuberth P., Nytko K.J., Pruschy M., Sulser S., Haefner M., Jensen S. (2018). Aberrant Lck Signal via CD28 Costimulation Augments Antigen-Specific Functionality and Tumor Control by Redirected T Cells with PD-1 Blockade in Humanized Mice. Clin. Cancer Res..

[167] John L.B., Devaud C., Duong C.P., Yong C.S., Beavis P.A., Haynes N.M., Chow M.T., Smyth M.J., Kershaw M.H., Darcy P.K. (2013). Anti-PD-1 antibody therapy potently enhances the eradication of established tumors by gene-modified T cells. Clin. Cancer Res..

[168] Serganova I., Moroz E., Cohen I., Moroz M., Mane M., Zurita J., Shenker L., Ponomarev V., Blasberg R. (2017). Enhancement of PSMA-Directed CAR Adoptive Immunotherapy by PD-1/PD-L1 Blockade. Mol. Ther. Oncolytics.

[169] Li H., Yuan W., Bin S., Wu G., Li P., Liu M., Yang J., Li X., Yang K., Gu H. (2020). Overcome trastuzumab resistance of breast cancer using anti-HER2 chimeric antigen receptor T cells and PD1 blockade. Am. J. Cancer Res..

[170] Kato D., Yaguchi T., Iwata T., Katoh Y., Morii K., Tsubota K., Takise Y., Tamiya M., Kamada H., Akiba H. (2020). GPC1 specific CAR-T cells eradicate established solid tumor without adverse effects and synergize with anti-PD-1 Ab. eLife.

[171] Song Y., Liu Q., Zuo T., Wei G., Jiao S. (2020). Combined antitumor effects of anti-EGFR variant III CAR-T cell therapy and PD-1 checkpoint blockade on glioblastoma in mouse model. Cell. Immunol..

[172] Tanoue K., Rosewell Shaw A., Watanabe N., Porter C., Rana B., Gottschalk S., Brenner M., Suzuki M. (2017). Armed Oncolytic Adenovirus-Expressing PD-L1 Mini-Body Enhances Antitumor Effects of Chimeric Antigen Receptor T Cells in Solid Tumors. Cancer Res..

[173] Heczey A., Louis C.U., Savoldo B., Dakhova O., Durett A., Grilley B., Liu H., Wu M.F., Mei Z., Gee A. (2017). CAR T Cells Administered in Combination with Lymphodepletion and PD-1 Inhibition to Patients with Neuroblastoma. Mol. Ther..

[174] Marotte L., Simon S., Vignard V., Dupre E., Gantier M., Cruard J., Alberge J.B., Hussong M., Deleine C., Heslan J.M. (2020). Increased antitumor efficacy of PD-1-deficient melanoma-specific human lymphocytes. J. Immunother. Cancer.

[175] Hu B., Zou Y., Zhang L., Tang J., Niedermann G., Firat E., Huang X., Zhu X. (2019). Nucleofection with Plasmid DNA for CRISPR/Cas9-Mediated Inactivation of Programmed Cell Death Protein 1 in CD133-Specific CAR T Cells. Hum. Gene Ther..

[176] Hu W., Zi Z., Jin Y., Li G., Shao K., Cai Q., Ma X., Wei F. (2019). CRISPR/Cas9-mediated PD-1 disruption enhances human mesothelin-targeted CAR T cell effector functions. Cancer Immunol. Immunother..

[177] Nakazawa T., Natsume A., Nishimura F., Morimoto T., Matsuda R., Nakamura M., Yamada S., Nakagawa I., Motoyama Y., Park Y.S. (2020). Effect of CRISPR/Cas9-Mediated PD-1-Disrupted Primary Human Third-Generation CAR-T Cells Targeting EGFRvIII on In Vitro Human Glioblastoma Cell Growth. Cells.

[178] Simon B., Harrer D.C., Schuler-Thurner B., Schaft N., Schuler G., Dorrie J., Uslu U. (2018). The siRNA-mediated downregulation of PD-1 alone or simultaneously with CTLA-4 shows enhanced in vitro CAR-T-cell functionality for further clinical development towards the potential use in immunotherapy of melanoma. Exp. Dermatol..

[179] Ren J., Liu X., Fang C., Jiang S., June C.H., Zhao Y. (2017). Multiplex Genome Editing to Generate Universal CAR T Cells Resistant to PD1 Inhibition. Clin. Cancer Res..

[180] Rupp L.J., Schumann K., Roybal K.T., Gate R.E., Ye C.J., Lim W.A., Marson A. (2017). CRISPR/Cas9-mediated PD-1 disruption enhances anti-tumor efficacy of human chimeric antigen receptor T cells. Sci. Rep..

[181] Zou F., Lu L., Liu J., Xia B., Zhang W., Hu Q., Liu W., Zhang Y., Lin Y., Jing S. (2019). Engineered triple inhibitory receptor resistance improves anti-tumor CAR-T cell performance via CD56. Nat. Commun..

[182] Shi X., Zhang D., Li F., Zhang Z., Wang S., Xuan Y., Ping Y., Zhang Y. (2019). Targeting glycosylation of PD-1 to enhance CAR-T cell cytotoxicity. J. Hematol. Oncol..

[183] Li X., Yao W., Yuan Y., Chen P., Li B., Li J., Chu R., Song H., Xie D., Jiang X. (2017). Targeting of tumour-infiltrating macrophages via CCL2/CCR2 signalling as a therapeutic strategy against hepatocellular carcinoma. Gut.

[184] Kuratsu J., Yoshizato K., Yoshimura T., Leonard E.J., Takeshima H., Ushio Y. (1993). Quantitative study of monocyte chemoattractant protein-1 (MCP-1) in cerebrospinal fluid and cyst fluid from patients with malignant glioma. J. Natl. Cancer Inst..

[185] Graves D.T., Barnhill R., Galanopoulos T., Antoniades H.N. (1992). Expression of monocyte chemotactic protein-1 in human melanoma in vivo. Am. J. Pathol..

[186] Shum T., Omer B., Tashiro H., Kruse R.L., Wagner D.L., Parikh K., Yi Z., Sauer T., Liu D., Parihar R. (2017). Constitutive Signaling from an Engineered IL7 Receptor Promotes Durable Tumor Elimination by Tumor-Redirected T Cells. Cancer Discov..

[187] Zhao Z., Li Y., Liu W., Li X. (2020). Engineered IL-7 Receptor Enhances the Therapeutic Effect of AXL-CAR-T Cells on Triple-Negative Breast Cancer. BioMed Res. Int..

[188] Weimin S., Abula A., Qianghong D., Wenguang W. (2020). Chimeric cytokine receptor enhancing PSMA-CAR-T cell-mediated prostate cancer regression. Cancer Biol. Ther..

[189] Mohammed S., Sukumaran S., Bajgain P., Watanabe N., Heslop H.E., Rooney C.M., Brenner M.K., Fisher W.E., Leen A.M., Vera J.F. (2017). Improving Chimeric Antigen Receptor-Modified T Cell Function by Reversing the Immunosuppressive Tumor Microenvironment of Pancreatic Cancer. Mol. Ther..

[190] Tan A.H.J., Vinanica N., Campana D. (2020). Chimeric antigen receptor-T cells with cytokine neutralizing capacity. Blood Adv..

[191] Wu X., Luo H., Shi B., Di S., Sun R., Su J., Liu Y., Li H., Jiang H., Li Z. (2019). Combined Antitumor Effects of Sorafenib and GPC3-CAR T Cells in Mouse Models of Hepatocellular Carcinoma. Mol. Ther..

[192] Wu X., Huang S. (2019). HER2-specific chimeric antigen receptor-engineered natural killer cells combined with apatinib for the treatment of gastric cancer. Bull. Cancer.

[193] Wang X., Walter M., Urak R., Weng L., Huynh C., Lim L., Wong C.W., Chang W.C., Thomas S.H., Sanchez J.F. (2018). Lenalidomide Enhances the Function of CS1 Chimeric Antigen Receptor-Redirected T Cells against Multiple Myeloma. Clin. Cancer Res..

[194] Torres-Collado A.X., Jazirehi A.R. (2018). Overcoming Resistance of Human Non-Hodgkin’s Lymphoma to CD19-CAR CTL Therapy by Celecoxib and Histone Deacetylase Inhibitors. Cancers.

[195] Zhang Q., Xu J., Ding J., Liu H., Li H., Li H., Lu M., Miao Y., Wang Z., Fu Q. (2018). Bortezomib improves adoptive carbonic anhydrase IXspecific chimeric antigen receptormodified NK92 cell therapy in mouse models of human renal cell carcinoma. Oncol. Rep..

[196] Charan M., Dravid P., Cam M., Audino A., Gross A.C., Arnold M.A., Roberts R.D., Cripe T.P., Pertsemlidis A., Houghton P.J. (2020). GD2-directed CAR-T cells in combination with HGF-targeted neutralizing antibody (AMG102) prevent primary tumor growth and metastasis in Ewing sarcoma. Int. J. Cancer.

[197] Chulanetra M., Morchang A., Sayour E., Eldjerou L., Milner R., Lagmay J., Cascio M., Stover B., Slayton W., Chaicumpa W. (2020). GD2 chimeric antigen receptor modified T cells in synergy with sub-toxic level of doxorubicin targeting osteosarcomas. Am. J. Cancer Res..

[198] Zhang Q., Tian K., Xu J., Zhang H., Li L., Fu Q., Chai D., Li H., Zheng J. (2017). Synergistic Effects of Cabozantinib and EGFR-Specific CAR-NK-92 Cells in Renal Cell Carcinoma. J. Immunol. Res..

[199] Stroncek D.F., Ren J., Lee D.W., Tran M., Frodigh S.E., Sabatino M., Khuu H., Merchant M.S., Mackall C.L. (2016). Myeloid cells in peripheral blood mononuclear cell concentrates inhibit the expansion of chimeric antigen receptor T cells. Cytotherapy.

[200] Davis R.J., Moore E.C., Clavijo P.E., Friedman J., Cash H., Chen Z., Silvin C., Van Waes C., Allen C. (2017). Anti-PD-L1 Efficacy Can Be Enhanced by Inhibition of Myeloid-Derived Suppressor Cells with a Selective Inhibitor of PI3Kdelta/gamma. Cancer Res..

[201] Sun L., Clavijo P.E., Robbins Y., Patel P., Friedman J., Greene S., Das R., Silvin C., Van Waes C., Horn L.A. (2019). Inhibiting myeloid-derived suppressor cell trafficking enhances T cell immunotherapy. JCI Insight.

[202] Clavijo P.E., Moore E.C., Chen J., Davis R.J., Friedman J., Kim Y., Van Waes C., Chen Z., Allen C.T. (2017). Resistance to CTLA-4 checkpoint inhibition reversed through selective elimination of granulocytic myeloid cells. Oncotarget.

[203] Di S., Zhou M., Pan Z., Sun R., Chen M., Jiang H., Shi B., Luo H., Li Z. (2019). Combined Adjuvant of Poly I:C Improves Antitumor Effects of CAR-T Cells. Front. Oncol..

[204] Long A.H., Highfill S.L., Cui Y., Smith J.P., Walker A.J., Ramakrishna S., El-Etriby R., Galli S., Tsokos M.G., Orentas R.J. (2016). Reduction of MDSCs with All-trans Retinoic Acid Improves CAR Therapy Efficacy for Sarcomas. Cancer Immunol. Res..

[205] Fultang L., Panetti S., Ng M., Collins P., Graef S., Rizkalla N., Booth S., Lenton R., Noyvert B., Shannon-Lowe C. (2019). MDSC targeting with Gemtuzumab ozogamicin restores T cell immunity and immunotherapy against cancers. EBioMedicine.

[206] Dominguez G.A., Condamine T., Mony S., Hashimoto A., Wang F., Liu Q., Forero A., Bendell J., Witt R., Hockstein N. (2017). Selective Targeting of Myeloid-Derived Suppressor Cells in Cancer Patients Using DS-8273a, an Agonistic TRAIL-R2 Antibody. Clin. Cancer Res..

[207] Fallah-Mehrjardi K., Mirzaei H.R., Masoumi E., Jafarzadeh L., Rostamian H., Khakpoor-Koosheh M., Alishah K., Noorbakhsh F., Hadjati J. (2020). Pharmacological targeting of immune checkpoint A2aR improves function of anti-CD19 CAR T cells in vitro. Immunol. Lett..

[208] Masoumi E., Jafarzadeh L., Mirzaei H.R., Alishah K., Fallah-Mehrjardi K., Rostamian H., Khakpoor-Koosheh M., Meshkani R., Noorbakhsh F., Hadjati J. (2020). Genetic and pharmacological targeting of A2a receptor improves function of anti-mesothelin CAR T cells. J. Exp. Clin. Cancer Res..

[209] Ohteki T., Parsons M., Zakarian A., Jones R.G., Nguyen L.T., Woodgett J.R., Ohashi P.S. (2000). Negative regulation of T cell proliferation and interleukin 2 production by the serine threonine kinase GSK-3. J. Exp. Med..

[210] Welsh G.I., Miyamoto S., Price N.T., Safer B., Proud C.G. (1996). T-cell activation leads to rapid stimulation of translation initiation factor eIF2B and inactivation of glycogen synthase kinase-3. J. Biol. Chem..

[211] Sengupta S., Katz S.C., Sengupta S., Sampath P. (2018). Glycogen synthase kinase 3 inhibition lowers PD-1 expression, promotes long-term survival and memory generation in antigen-specific CAR-T cells. Cancer Lett..

[212] Klebanoff C.A., Crompton J.G., Leonardi A.J., Yamamoto T.N., Chandran S.S., Eil R.L., Sukumar M., Vodnala S.K., Hu J., Ji Y. (2017). Inhibition of AKT signaling uncouples T cell differentiation from expansion for receptor-engineered adoptive immunotherapy. JCI Insight.

[213] Zhang Q., Ding J., Sun S., Liu H., Lu M., Wei X., Gao X., Zhang X., Fu Q., Zheng J. (2019). Akt inhibition at the initial stage of CAR-T preparation enhances the CAR-positive expression rate, memory phenotype and in vivo efficacy. Am. J. Cancer Res..

[214] Kagoya Y., Nakatsugawa M., Yamashita Y., Ochi T., Guo T., Anczurowski M., Saso K., Butler M.O., Arrowsmith C.H., Hirano N. (2016). BET bromodomain inhibition enhances T cell persistence and function in adoptive immunotherapy models. J. Clin. Investig..

[215] Dwyer C.J., Arhontoulis D.C., Rangel Rivera G.O., Knochelmann H.M., Smith A.S., Wyatt M.M., Rubinstein M.P., Atkinson C., Thaxton J.E., Neskey D.M. (2020). Ex vivo blockade of PI3K gamma or delta signaling enhances the antitumor potency of adoptively transferred CD8(+) T cells. Eur. J. Immunol..

[216] Deng C., Zhao J., Zhou S., Dong J., Cao J., Gao J., Bai Y., Deng H. (2020). The Vascular Disrupting Agent CA4P Improves the Antitumor Efficacy of CAR-T Cells in Preclinical Models of Solid Human Tumors. Mol. Ther..

[217] Bocca P., Di Carlo E., Caruana I., Emionite L., Cilli M., De Angelis B., Quintarelli C., Pezzolo A., Raffaghello L., Morandi F. (2017). Bevacizumab-mediated tumor vasculature remodelling improves tumor infiltration and antitumor efficacy of GD2-CAR T cells in a human neuroblastoma preclinical model. Oncoimmunology.

[218] Fraietta J.A., Beckwith K.A., Patel P.R., Ruella M., Zheng Z., Barrett D.M., Lacey S.F., Melenhorst J.J., McGettigan S.E., Cook D.R. (2016). Ibrutinib enhances chimeric antigen receptor T-cell engraftment and efficacy in leukemia. Blood.

[219] Gauthier J., Hirayama A.V., Purushe J., Hay K.A., Lymp J., Li D.H., Yeung C.C.S., Sheih A., Pender B.S., Hawkins R.M. (2020). Feasibility and efficacy of CD19-targeted CAR T cells with concurrent ibrutinib for CLL after ibrutinib failure. Blood.

[220] Zhang H., Hu Y., Chang A.H., Huang H. (2020). Successful treatment of T315I BCR-ABL mutated lymphoid blast phase chronic myeloid leukemia with chimeric antigen receptor T cell therapy followed by dasatinib. Regen. Ther..

[221] Li S., Xue L., Wang M., Qiang P., Xu H., Zhang X., Kang W., You F., Xu H., Wang Y. (2019). Decitabine enhances cytotoxic effect of T cells with an anti-CD19 chimeric antigen receptor in treatment of lymphoma. OncoTargets Ther..

[222] Chen R., Wang M., Liu Q., Wu J., Huang W., Li X., Du B., Xu Q., Duan J., Jiao S. (2020). Sequential treatment with aT19cells generates memory CAR-T cells and prolongs the lifespan of Raji-B-NDG mice. Cancer Lett..

[223] Ivics Z. (2020). Potent CAR-T cells engineered with Sleeping Beauty transposon vectors display a central memory phenotype. Gene Ther..

[224] Urak R., Walter M., Lim L., Wong C.W., Budde L.E., Thomas S., Forman S.J., Wang X. (2017). Ex vivo Akt inhibition promotes the generation of potent CD19CAR T cells for adoptive immunotherapy. J. Immunother. Cancer.

[225] Petersen C.T., Hassan M., Morris A.B., Jeffery J., Lee K., Jagirdar N., Staton A.D., Raikar S.S., Spencer H.T., Sulchek T. (2018). Improving T-cell expansion and function for adoptive T-cell therapy using ex vivo treatment with PI3Kdelta inhibitors and VIP antagonists. Blood Adv..

[226] Stock S., Ubelhart R., Schubert M.L., Fan F., He B., Hoffmann J.M., Wang L., Wang S., Gong W., Neuber B. (2019). Idelalisib for optimized CD19-specific chimeric antigen receptor T cells in chronic lymphocytic leukemia patients. Int. J. Cancer.

[227] Zhang Z., Li F., Tian Y., Cao L., Gao Q., Zhang C., Zhang K., Shen C., Ping Y., Maimela N.R. (2020). Metformin Enhances the Antitumor Activity of CD8(+) T Lymphocytes via the AMPK-miR-107-Eomes-PD-1 Pathway. J. Immunol..

[228] Huang Y., Goel S., Duda D.G., Fukumura D., Jain R.K. (2013). Vascular normalization as an emerging strategy to enhance cancer immunotherapy. Cancer Res..

[229] Shrimali R.K., Yu Z., Theoret M.R., Chinnasamy D., Restifo N.P., Rosenberg S.A. (2010). Antiangiogenic agents can increase lymphocyte infiltration into tumor and enhance the effectiveness of adoptive immunotherapy of cancer. Cancer Res..

[230] Straathof K.C., Pule M.A., Yotnda P., Dotti G., Vanin E.F., Brenner M.K., Heslop H.E., Spencer D.M., Rooney C.M. (2005). An inducible caspase 9 safety switch for T-cell therapy. Blood.

[231] Fan L., Freeman K.W., Khan T., Pham E., Spencer D.M. (1999). Improved artificial death switches based on caspases and FADD. Hum. Gene Ther..

[232] Narayanan P., Lapteva N., Seethammagari M., Levitt J.M., Slawin K.M., Spencer D.M. (2011). A composite MyD88/CD40 switch synergistically activates mouse and human dendritic cells for enhanced antitumor efficacy. J. Clin. Investig..

[233] Wu C.Y., Roybal K.T., Puchner E.M., Onuffer J., Lim W.A. (2015). Remote control of therapeutic T cells through a small molecule-gated chimeric receptor. Science.

[234] Foster A.E., Mahendravada A., Shinners N.P., Chang W.C., Crisostomo J., Lu A., Khalil M., Morschl E., Shaw J.L., Saha S. (2017). Regulated Expansion and Survival of Chimeric Antigen Receptor-Modified T Cells Using Small Molecule-Dependent Inducible MyD88/CD40. Mol. Ther..

[235] Juillerat A., Marechal A., Filhol J.M., Valton J., Duclert A., Poirot L., Duchateau P. (2016). Design of chimeric antigen receptors with integrated controllable transient functions. Sci. Rep..

[236] Duong M.T., Collinson-Pautz M.R., Morschl E., Lu A., Szymanski S.P., Zhang M., Brandt M.E., Chang W.C., Sharp K.L., Toler S.M. (2019). Two-Dimensional Regulation of CAR-T Cell Therapy with Orthogonal Switches. Mol. Ther. Oncolytics.

[237] Leung W.H., Gay J., Martin U., Garrett T.E., Horton H.M., Certo M.T., Blazar B.R., Morgan R.A., Gregory P.D., Jarjour J. (2019). Sensitive and adaptable pharmacological control of CAR T cells through extracellular receptor dimerization. JCI Insight.

[238] Lee S.M., Kang C.H., Choi S.U., Kim Y., Hwang J.Y., Jeong H.G., Park C.H. (2020). A Chemical Switch System to Modulate Chimeric Antigen Receptor T Cell Activity through Proteolysis-Targeting Chimaera Technology. ACS Synth. Biol..

[239] Juillerat A., Tkach D., Busser B.W., Temburni S., Valton J., Duclert A., Poirot L., Depil S., Duchateau P. (2019). Modulation of chimeric antigen receptor surface expression by a small molecule switch. BMC Biotechnol..

[240] Roellecke K., Virts E.L., Einholz R., Edson K.Z., Altvater B., Rossig C., von Laer D., Scheckenbach K., Wagenmann M., Reinhardt D. (2016). Optimized human CYP4B1 in combination with the alkylator prodrug 4-ipomeanol serves as a novel suicide gene system for adoptive T-cell therapies. Gene Ther..

[241] Lu Y.J., Chu H., Wheeler L.W., Nelson M., Westrick E., Matthaei J.F., Cardle I.I., Johnson A., Gustafson J., Parker N. (2019). Preclinical Evaluation of Bispecific Adaptor Molecule Controlled Folate Receptor CAR-T Cell Therapy with Special Focus on Pediatric Malignancies. Front. Oncol..

[242] Kim M.S., Ma J.S., Yun H., Cao Y., Kim J.Y., Chi V., Wang D., Woods A., Sherwood L., Caballero D. (2015). Redirection of genetically engineered CAR-T cells using bifunctional small molecules. J. Am. Chem. Soc..

[243] Futami M., Suzuki K., Kato S., Ohmae S., Tahara Y., Nojima M., Imai Y., Mimura T., Watanabe Y., Tojo A. (2020). The novel multi-cytokine inhibitor TO-207 specifically inhibits pro-inflammatory cytokine secretion in monocytes without affecting the killing ability of CAR T cells. PLoS ONE.

[244] Mestermann K., Giavridis T., Weber J., Rydzek J., Frenz S., Nerreter T., Mades A., Sadelain M., Einsele H., Hudecek M. (2019). The tyrosine kinase inhibitor dasatinib acts as a pharmacologic on/off switch for CAR T cells. Sci. Transl. Med..

[245] Weber E.W., Lynn R.C., Sotillo E., Lattin J., Xu P., Mackall C.L. (2019). Pharmacologic control of CAR-T cell function using dasatinib. Blood Adv..

[246] Song D.G., Ye Q., Santoro S., Fang C., Best A., Powell D.J. (2013). Chimeric NKG2D CAR-expressing T cell-mediated attack of human ovarian cancer is enhanced by histone deacetylase inhibition. Hum. Gene Ther..

[247] Kailayangiri S., Altvater B., Lesch S., Balbach S., Gottlich C., Kuhnemundt J., Mikesch J.H., Schelhaas S., Jamitzky S., Meltzer J. (2019). EZH2 Inhibition in Ewing Sarcoma Upregulates GD2 Expression for Targeting with Gene-Modified T Cells. Mol. Ther..

[248] Mihara K., Yoshida T., Ishida S., Takei Y., Kitanaka A., Shimoda K., Morishita K., Takihara Y., Ichinohe T. (2016). All-trans retinoic acid and interferon-alpha increase CD38 expression on adult T-cell leukemia cells and sensitize them to T cells bearing anti-CD38 chimeric antigen receptors. Blood Cancer J..

[249] Xu Y., Li S., Wang Y., Liu J., Mao X., Xing H., Tian Z., Tang K., Liao X., Rao Q. (2019). Induced CD20 Expression on B-Cell Malignant Cells Heightened the Cytotoxic Activity of Chimeric Antigen Receptor Engineered T Cells. Hum. Gene Ther..

[250] Ramakrishna S., Highfill S.L., Walsh Z., Nguyen S.M., Lei H., Shern J.F., Qin H., Kraft I.L., Stetler-Stevenson M., Yuan C.M. (2019). Modulation of Target Antigen Density Improves CAR T-cell Functionality and Persistence. Clin. Cancer Res..

[251] Klar A.S., Gopinadh J., Kleber S., Wadle A., Renner C. (2015). Treatment with 5-Aza-2′-Deoxycytidine Induces Expression of NY-ESO-1 and Facilitates Cytotoxic T Lymphocyte-Mediated Tumor Cell Killing. PLoS ONE.

[252] Shiozawa M., Chang C.H., Huang Y.C., Chen Y.C., Chi M.S., Hao H.C., Chang Y.C., Takeda S., Chi K.H., Wang Y.S. (2018). Pharmacologically upregulated carcinoembryonic antigen-expression enhances the cytolytic activity of genetically-modified chimeric antigen receptor NK-92MI against colorectal cancer cells. BMC Immunol..

[253] Hardman C., Ho S., Shimizu A., Luu-Nguyen Q., Sloane J.L., Soliman M.S.A., Marsden M.D., Zack J.A., Wender P.A. (2020). Synthesis and evaluation of designed PKC modulators for enhanced cancer immunotherapy. Nat. Commun..

[254] Bao L., Dunham K., Lucas K. (2011). MAGE-A1, MAGE-A3, and NY-ESO-1 can be upregulated on neuroblastoma cells to facilitate cytotoxic T lymphocyte-mediated tumor cell killing. Cancer Immunol. Immunother..

[255] Jetani H., Garcia-Cadenas I., Nerreter T., Thomas S., Rydzek J., Meijide J.B., Bonig H., Herr W., Sierra J., Einsele H. (2018). CAR T-cells targeting FLT3 have potent activity against FLT3(-)ITD(+) AML and act synergistically with the FLT3-inhibitor crenolanib. Leukemia.

[256] Li H., Ding J., Lu M., Liu H., Miao Y., Li L., Wang G., Zheng J., Pei D., Zhang Q. (2020). CAIX-specific CAR-T Cells and Sunitinib Show Synergistic Effects Against Metastatic Renal Cancer Models. J. Immunother..

[257] Akahori Y., Wang L., Yoneyama M., Seo N., Okumura S., Miyahara Y., Amaishi Y., Okamoto S., Mineno J., Ikeda H. (2018). Antitumor activity of CAR-T cells targeting the intracellular oncoprotein WT1 can be enhanced by vaccination. Blood.

[258] Tanaka M., Tashiro H., Omer B., Lapteva N., Ando J., Ngo M., Mehta B., Dotti G., Kinchington P.R., Leen A.M. (2017). Vaccination Targeting Native Receptors to Enhance the Function and Proliferation of Chimeric Antigen Receptor (CAR)-Modified T Cells. Clin. Cancer Res..

[259] Slaney C.Y., von Scheidt B., Davenport A.J., Beavis P.A., Westwood J.A., Mardiana S., Tscharke D.C., Ellis S., Prince H.M., Trapani J.A. (2017). Dual-specific Chimeric Antigen Receptor T Cells and an Indirect Vaccine Eradicate a Variety of Large Solid Tumors in an Immunocompetent, Self-antigen Setting. Clin. Cancer Res..

[260] Rossig C., Pule M., Altvater B., Saiagh S., Wright G., Ghorashian S., Clifton-Hadley L., Champion K., Sattar Z., Popova B. (2017). Vaccination to improve the persistence of CD19CAR gene-modified T cells in relapsed pediatric acute lymphoblastic leukemia. Leukemia.

[261] Ma L., Dichwalkar T., Chang J.Y.H., Cossette B., Garafola D., Zhang A.Q., Fichter M., Wang C., Liang S., Silva M. (2019). Enhanced CAR-T cell activity against solid tumors by vaccine boosting through the chimeric receptor. Science.

[262] Reinhard K., Rengstl B., Oehm P., Michel K., Billmeier A., Hayduk N., Klein O., Kuna K., Ouchan Y., Woll S. (2020). An RNA vaccine drives expansion and efficacy of claudin-CAR-T cells against solid tumors. Science.

[263] Smith E.L., Mailankody S., Staehr M., Wang X., Senechal B., Purdon T.J., Daniyan A.F., Geyer M.B., Goldberg A.D., Mead E. (2019). BCMA-Targeted CAR T-cell Therapy plus Radiotherapy for the Treatment of Refractory Myeloma Reveals Potential Synergy. Cancer Immunol. Res..

[264] Weiss T., Weller M., Guckenberger M., Sentman C.L., Roth P. (2018). NKG2D-Based CAR T Cells and Radiotherapy Exert Synergistic Efficacy in Glioblastoma. Cancer Res..

[265] DeSelm C., Palomba M.L., Yahalom J., Hamieh M., Eyquem J., Rajasekhar V.K., Sadelain M. (2018). Low-Dose Radiation Conditioning Enables CAR T Cells to Mitigate Antigen Escape. Mol. Ther..

[266] Qu C., Ping N., Kang L., Liu H., Qin S., Wu Q., Chen X., Zhou M., Xia F., Ye A. (2020). Radiation Priming Chimeric Antigen Receptor T-Cell Therapy in Relapsed/Refractory Diffuse Large B-Cell Lymphoma with High Tumor Burden. J. Immunother..

[267] Sim A.J., Jain M.D., Figura N.B., Chavez J.C., Shah B.D., Khimani F., Lazaryan A., Krivenko G., Davila M.L., Liu H.D. (2019). Radiation Therapy as a Bridging Strategy for CAR T Cell Therapy with Axicabtagene Ciloleucel in Diffuse Large B-Cell Lymphoma. Int. J. Radiat. Oncol. Biol. Phys..

[268] Wright C.M., LaRiviere M.J., Baron J.A., Uche C., Xiao Y., Arscott W.T., Anstadt E.J., Barsky A.R., Miller D., LaRose M.I. (2020). Bridging Radiation Therapy Prior to Commercial Chimeric Antigen Receptor T-Cell Therapy for relapsed/refractory aggressive B-cell lymphoma. Int. J. Radiat. Oncol. Biol. Phys..

[269] Imber B.S., Sadelain M., DeSelm C., Batlevi C., Brentjens R.J., Dahi P.B., Giralt S., Park J.H., Sauter C., Scordo M. (2020). Early experience using salvage radiotherapy for relapsed/refractory non-Hodgkin lymphomas after CD19 chimeric antigen receptor (CAR) T cell therapy. Br. J. Haematol..

[270] Jiang Y.L., Li Q., Yuan T., Jiang Y.Y., Deng Q. (2020). Case Report of Anti-CD123 Chimeric Antigen Receptor T-Cell Therapy Followed by Radiotherapy for a Recurrence of Blastic Plasmacytoid Dendritic Cell Neoplasm After Allogeneic Hematopoietic Stem Cell Transplantation. OncoTargets Ther..

[271] Long M., Beckwith K., Do P., Mundy B.L., Gordon A., Lehman A.M., Maddocks K.J., Cheney C., Jones J.A., Flynn J.M. (2017). Ibrutinib treatment improves T cell number and function in CLL patients. J. Clin. Investig..

[272] Ruella M., Kenderian S.S., Shestova O., Klichinsky M., Melenhorst J.J., Wasik M.A., Lacey S.F., June C.H., Gill S. (2017). Kinase inhibitor ibrutinib to prevent cytokine-release syndrome after anti-CD19 chimeric antigen receptor T cells for B-cell neoplasms. Leukemia.

[273] Allen E.S., Stroncek D.F., Ren J., Eder A.F., West K.A., Fry T.J., Lee D.W., Mackall C.L., Conry-Cantilena C. (2017). Autologous lymphapheresis for the production of chimeric antigen receptor T cells. Transfusion.

[274] Elavia N., Panch S.R., McManus A., Bikkani T., Szymanski J., Highfill S.L., Jin P., Brudno J., Kochenderfer J., Stroncek D.F. (2019). Effects of starting cellular material composition on chimeric antigen receptor T-cell expansion and characteristics. Transfusion.

[275] Das R.K., Vernau L., Grupp S.A., Barrett D.M. (2019). Naive T-cell Deficits at Diagnosis and after Chemotherapy Impair Cell Therapy Potential in Pediatric Cancers. Cancer Discov..

[276] Barrett D.M., Singh N., Liu X., Jiang S., June C.H., Grupp S.A., Zhao Y. (2014). Relation of clinical culture method to T-cell memory status and efficacy in xenograft models of adoptive immunotherapy. Cytotherapy.

[277] Kagoya Y., Nakatsugawa M., Ochi T., Cen Y., Guo T., Anczurowski M., Saso K., Butler M.O., Hirano N. (2017). Transient stimulation expands superior antitumor T cells for adoptive therapy. JCI Insight.

[278] Shrestha B., Zhang Y., Yu B., Li G., Boucher J.C., Beatty N.J., Tsai H.C., Wang X., Mishra A., Sweet K. (2020). Generation of Antitumor T Cells For Adoptive Cell Therapy With Artificial Antigen Presenting Cells. J. Immunother..

[279] Kebriaei P., Singh H., Huls M.H., Figliola M.J., Bassett R., Olivares S., Jena B., Dawson M.J., Kumaresan P.R., Su S. (2016). Phase I trials using Sleeping Beauty to generate CD19-specific CAR T cells. J. Clin. Investig..

[280] Zabel M., Tauber P.A., Pickl W.F. (2019). The making and function of CAR cells. Immunol. Lett..

[281] Xu Y., Zhang M., Ramos C.A., Durett A., Liu E., Dakhova O., Liu H., Creighton C.J., Gee A.P., Heslop H.E. (2014). Closely related T-memory stem cells correlate with in vivo expansion of CAR.CD19-T cells and are preserved by IL-7 and IL-15. Blood.

[282] Kondo T., Imura Y., Chikuma S., Hibino S., Omata-Mise S., Ando M., Akanuma T., Iizuka M., Sakai R., Morita R. (2018). Generation and application of human induced-stem cell memory T cells for adoptive immunotherapy. Cancer Sci..

[283] Cieri N., Camisa B., Cocchiarella F., Forcato M., Oliveira G., Provasi E., Bondanza A., Bordignon C., Peccatori J., Ciceri F. (2013). IL-7 and IL-15 instruct the generation of human memory stem T cells from naive precursors. Blood.

[284] Gong W., Hoffmann J.M., Stock S., Wang L., Liu Y., Schubert M.L., Neuber B., Huckelhoven-Krauss A., Gern U., Schmitt A. (2019). Comparison of IL-2 vs IL-7/IL-15 for the generation of NY-ESO-1-specific T cells. Cancer Immunol. Immunother..

[285] Marcucci K.T., Jadlowsky J.K., Hwang W.T., Suhoski-Davis M., Gonzalez V.E., Kulikovskaya I., Gupta M., Lacey S.F., Plesa G., Chew A. (2018). Retroviral and Lentiviral Safety Analysis of Gene-Modified T Cell Products and Infused HIV and Oncology Patients. Mol. Ther..

[286] Wang P., Qin W., Liu T., Jiang D., Cui L., Liu X., Fang Y., Tang X., Jin H., Qian Q. (2020). PiggyBac-engineered T cells expressing a glypican-3-specific chimeric antigen receptor show potent activities against hepatocellular carcinoma. Immunobiology.

[287] Chicaybam L., Abdo L., Viegas M., Marques L.V.C., de Sousa P., Batista-Silva L.R., Alves-Monteiro V., Bonecker S., Monte-Mor B., Bonamino M.H. (2020). Transposon-mediated generation of CAR-T cells shows efficient anti B-cell leukemia response after ex vivo expansion. Gene Ther..

[288] Monjezi R., Miskey C., Gogishvili T., Schleef M., Schmeer M., Einsele H., Ivics Z., Hudecek M. (2017). Enhanced CAR T-cell engineering using non-viral Sleeping Beauty transposition from minicircle vectors. Leukemia.

[289] Xu J.Y., Ye Z.L., Jiang D.Q., He J.C., Ding Y.M., Li L.F., Lv S.Q., Wang Y., Jin H.J., Qian Q.J. (2017). Mesothelin-targeting chimeric antigen receptor-modified T cells by piggyBac transposon system suppress the growth of bile duct carcinoma. Tumour Biol..

[290] Srour S.A., Singh H., McCarty J., de Groot E., Huls H., Rondon G., Qazilbash M., Ciurea S., Bardelli G., Buck J. (2020). Long-term outcomes of Sleeping Beauty-generated CD19-specific CAR T-cell therapy for relapsed-refractory B-cell lymphomas. Blood.

[291] Tu S., Huang R., Guo Z., Deng L., Song C., Zhou X., Yue C., Zhang L., He Y., Yang J. (2019). Shortening the ex vivo culture of CD19-specific CAR T-cells retains potent efficacy against acute lymphoblastic leukemia without CAR T-cell-related encephalopathy syndrome or severe cytokine release syndrome. Am. J. Hematol..

[292] Caruso H.G., Tanaka R., Liang J., Ling X., Sabbagh A., Henry V.K., Collier T.L., Heimberger A.B. (2019). Shortened ex vivo manufacturing time of EGFRvIII-specific chimeric antigen receptor (CAR) T cells reduces immune exhaustion and enhances antiglioma therapeutic function. J. Neuro-Oncol..

[293] Ghassemi S., Nunez-Cruz S., O’Connor R.S., Fraietta J.A., Patel P.R., Scholler J., Barrett D.M., Lundh S.M., Davis M.M., Bedoya F. (2018). Reducing Ex Vivo Culture Improves the Antileukemic Activity of Chimeric Antigen Receptor (CAR) T Cells. Cancer Immunol. Res..

[294] Kaartinen T., Luostarinen A., Maliniemi P., Keto J., Arvas M., Belt H., Koponen J., Makinen P.I., Loskog A., Mustjoki S. (2017). Low interleukin-2 concentration favors generation of early memory T cells over effector phenotypes during chimeric antigen receptor T-cell expansion. Cytotherapy.

[295] Zhang X., Lv X., Song Y. (2018). Short-term culture with IL-2 is beneficial for potent memory chimeric antigen receptor T cell production. Biochem. Biophys. Res. Commun..

[296] Finney O.C., Brakke H.M., Rawlings-Rhea S., Hicks R., Doolittle D., Lopez M., Futrell R.B., Orentas R.J., Li D., Gardner R.A. (2019). CD19 CAR T cell product and disease attributes predict leukemia remission durability. J. Clin. Investig..

[297] Panch S.R., Srivastava S.K., Elavia N., McManus A., Liu S., Jin P., Highfill S.L., Li X., Dagur P., Kochenderfer J.N. (2019). Effect of Cryopreservation on Autologous Chimeric Antigen Receptor T Cell Characteristics. Mol. Ther..

[298] Hanley P.J. (2019). Fresh versus Frozen: Effects of Cryopreservation on CAR T Cells. Mol. Ther..

[299] Tyagarajan S., Schmitt D., Acker C., Rutjens E. (2019). Autologous cryopreserved leukapheresis cellular material for chimeric antigen receptor-T cell manufacture. Cytotherapy.

[300] Xu H., Cao W., Huang L., Xiao M., Cao Y., Zhao L., Wang N., Zhou J. (2018). Effects of cryopreservation on chimeric antigen receptor T cell functions. Cryobiology.

[301] Park A.K., Fong Y., Kim S.I., Yang J., Murad J.P., Lu J., Jeang B., Chang W.C., Chen N.G., Thomas S.H. (2020). Effective combination immunotherapy using oncolytic viruses to deliver CAR targets to solid tumors. Sci. Transl. Med..

[302] Klichinsky M., Ruella M., Shestova O., Lu X.M., Best A., Zeeman M., Schmierer M., Gabrusiewicz K., Anderson N.R., Petty N.E. (2020). Human chimeric antigen receptor macrophages for cancer immunotherapy. Nat. Biotechnol..

[303] Benjamin R., Graham C., Yallop D., Jozwik A., Mirci-Danicar O.C., Lucchini G., Pinner D., Jain N., Kantarjian H., Boissel N. (2020). Genome-edited, donor-derived allogeneic anti-CD19 chimeric antigen receptor T cells in paediatric and adult B-cell acute lymphoblastic leukaemia: Results of two phase 1 studies. Lancet.

[304] Liu E., Marin D., Banerjee P., Macapinlac H.A., Thompson P., Basar R., Nassif Kerbauy L., Overman B., Thall P., Kaplan M. (2020). Use of CAR-Transduced Natural Killer Cells in CD19-Positive Lymphoid Tumors. N. Engl. J. Med..

